# StARD9 is a novel lysosomal kinesin required for membrane tubulation, cholesterol transport and Purkinje cell survival

**DOI:** 10.1242/jcs.260662

**Published:** 2023-03-02

**Authors:** Felicity R. Sterling, Jon D'Amico, Alexandria M. Brumfield, Kara L. Huegel, Patricia S. Vaughan, Kathryn Morris, Shelby Schwarz, Michelle V. Joyce, Bill Boggess, Matthew M. Champion, Kevin Maciuba, Philip Allen, Eric Marasco, Grant Koch, Peter Gonzalez, Shannon Hodges, Shannon Leahy, Erica Gerstbauer, Edward H. Hinchcliffe, Kevin T. Vaughan

**Affiliations:** ^1^ Department of Biological Sciences, University of Notre Dame, Notre Dame, IN 46556, USA; ^2^ Department of Chemistry and Biochemistry, University of Notre Dame, Notre Dame, IN 46556, USA; ^3^ University of Notre Dame Proteomics and Mass Spectrometry Facility, University of Notre Dame, Notre Dame, IN 46556, USA; ^4^ Hormel Institute, University of Minnesota, Austin, MN 55912, USA; ^5^ Notre Dame Integrated Imaging Facility, University of Notre Dame, Notre Dame, IN 46556, USA

**Keywords:** Kinesin, Cholesterol, Lysosome, Niemann–Pick type C disease, Membrane tubulation

## Abstract

The pathological accumulation of cholesterol is a signature feature of Niemann–Pick type C (NPC) disease, in which excessive lipid levels induce Purkinje cell death in the cerebellum. NPC1 encodes a lysosomal cholesterol-binding protein, and mutations in NPC1 drive cholesterol accumulation in late endosomes and lysosomes (LE/Ls). However, the fundamental role of NPC proteins in LE/L cholesterol transport remains unclear. Here, we demonstrate that NPC1 mutations impair the projection of cholesterol-containing membrane tubules from the surface of LE/Ls. A proteomic survey of purified LE/Ls identified StARD9 as a novel lysosomal kinesin responsible for LE/L tubulation. StARD9 contains an N-terminal kinesin domain, a C-terminal StART domain, and a dileucine signal shared with other lysosome-associated membrane proteins. Depletion of StARD9 disrupts LE/L tubulation, paralyzes bidirectional LE/L motility and induces accumulation of cholesterol in LE/Ls. Finally, a novel StARD9 knock-out mouse recapitulates the progressive loss of Purkinje cells in the cerebellum. Together, these studies identify StARD9 as a microtubule motor protein responsible for LE/L tubulation and provide support for a novel model of LE/L cholesterol transport that becomes impaired in NPC disease.

## Teaser

**Summary:** StARD9 is a novel lysosomal kinesin that functions with genes implicated in Niemann–Pick type C disease to drive lysosomal tubulation. The loss of tubulation is proposed as a defect in Niemann–Pick type C disease.

## INTRODUCTION

Late endosomes and lysosomes (LE/Ls) are catabolic organelles in eukaryotes that are responsible for processing materials acquired by endocytosis, autophagy and phagocytosis for utilization and survival. Lysosomal storage diseases (LSDs) are a class of inherited diseases in which one or more components of LE/Ls are impaired (Cox and Cachón-González, 2012; Parenti et al., 2021). Genetic mutations in catabolic enzyme genes define a majority of LSDs (Solomon and Muro, 2017). However, complicating our search for treatments, sometimes the LSD is caused by mutations in genes that do not encode a catabolic enzyme.

Niemann–Pick type C (NPC) disease is a pediatric neurodegenerative disorder caused by the pathological accumulation of cholesterol and other lipids in LE/Ls (Ory, 2000). Although cholesterol accumulation is apparent in the liver and spleen, the primary problem in NPC disease is the progressive loss of Purkinje cells in the cerebellum (Higashi et al., 1993; Võikar et al., 2002). This loss of Purkinje cells correlates well with the development of ataxia, tremors, slurred speech, loss of eye control and seizures (Sturley et al., 2004). Although several treatment strategies are under investigation (Pineda et al., 2009; Chandler et al., 2017; Ory et al., 2017), no treatment for NPC disease has yet received approval from the US Food and Drug Administration (FDA).

Two genes (*NPC1* and *NPC2*) have been implicated in causing NPC disease (Carstea et al., 1997; Naureckiene et al., 2000; Liscum and Sturley, 2004). NPC1 is a multi-pass transmembrane protein that resides in LE/Ls, and mutations in NPC1 are responsible for ∼95% of NPC disease cases (Neufeld et al., 1999; Davies and Ioannou, 2000; Infante et al., 2008a,b). It contains an N-terminal cholesterol-binding domain (Infante et al., 2008a,b; Kwon et al., 2009) and a transmembrane sterol-sensing domain (SSD) (Ohgane et al., 2013). NPC2 is a soluble LE/L protein thought to bind cholesterol, and mutations in NPC2 are responsible for the remaining 5% of cases (Xu et al., 2007).

Although both NPC1 and NPC2 have cholesterol-binding domains, they lack signatures of additional functions. No enzymatic activities have been identified for these proteins nor do they show indicators of ion binding, nucleotide binding or molecular motor function. The predicted orientation of cholesterol in the binding pockets of NPC1 and NPC2 suggests that the two could exchange cholesterol between each other in a ‘hand-off’ mechanism (Infante et al., 2008a,b). Structural studies of NPC1 also suggest that the SSD forms a transmembrane tunnel providing a path for cholesterol across the LE/L limiting membrane (Li et al., 2016; Qian et al., 2020). Furthermore, the U18666A drug used to mimic NPC disease blocks this tunnel (Li et al., 2016), providing an explanation for how this drug works and the importance of the SSD. Given the hydrophobic nature of cholesterol, the ultimate destination of this steroid remains unclear after it crosses the LE/L limiting membrane.

Complicating our search for effective therapies, little consensus exists for the specific biological process that leads to lipid accumulation in NPC disease. Although NPC1 and NPC2 both bind cholesterol, the lipid accumulations in NPC disease include cholesterol, oxysterols, sphingolipids, gangliosides and glycosylphosphatidylinositol (GPI)-anchored proteins (Zervas et al., 2001; Lloyd-Evans and Platt, 2010). This suggests that some function more global than cholesterol transport alone is impaired. Ca^2+^ levels and membrane excitability are also affected (Tiscione et al., 2019). However, neither NPC1 nor NPC2 contain Ca^2+^-binding domains. Because mutations in NPC1 do not concentrate in any one domain, NPC disease has been identified as a protein-folding disease. Thorough analysis of the NPC1 I1061T mutation supports this model (Gelsthorpe et al., 2008; Schultz et al., 2018), implicating loss of protein accumulation as the defect. The impact of NPC1 mutations on the thickness of the glycocalyx in LE/Ls suggests that NPC1 serves a lysosome-associated membrane protein (LAMP) function as well (Li et al., 2015). Finally, defects in the delivery of cholesterol to the endoplasmic reticulum (Höglinger et al., 2019) and plasma membrane (Kanerva et al., 2013) suggest a role for NPC proteins in LE/L cholesterol efflux.

In an effort to identify a promising therapy for NPC disease, we sought a global function for NPC1 and NPC2 that could explain the complexities of this disease. We identified LE/L tubulation as a fundamental mechanism of lipid transport, which is impaired in NPC disease. We also identified StARD9 as a novel LE/L kinesin responsible for LE/L tubulation. StARD9 colocalized with NPC1 and depletion of StARD9 induced the same defects in LE/L transport as NPC1 depletion. Finally, StARD9 knock-out (KO) mice developed the same progressive loss of Purkinje cells and development of neurodegeneration as NPC1 mutant mice. We propose that loss of LE/L tubulation is a significant defect in NPC disease and that NPC1 functions with StARD9 to drive LE/L tubulation.

## RESULTS

### NPC disease and lysosomal tubulation

**Fig. 1.**
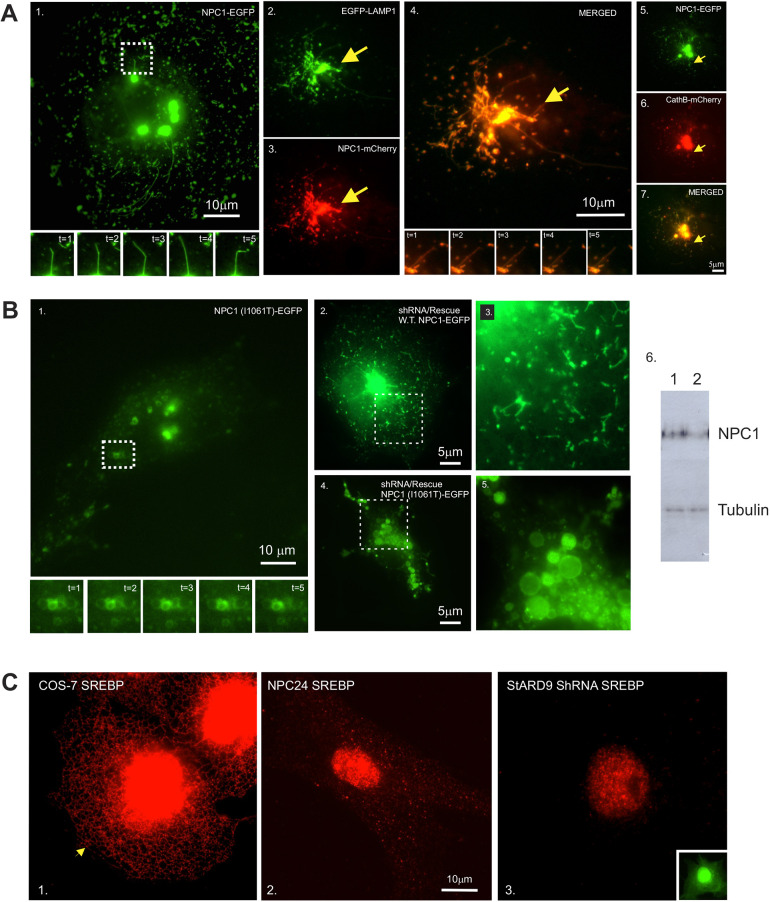
**Lysosomal tubulation in cells expressing wild-type or mutant NPC1.** (A) COS-7 cells transfected with wild-type NPC1–EGFP alone (panel 1), EGFP–LAMP1 and NPC1–mCherry (panels 2–4), NPC1–EGFP and cathepsin B (CathB)–mCherry (panels 5–7) display projection of dynamic membrane tubules from LE/Ls. Yellow arrows identify membrane tubules. Time-lapse image sequences depict 1 s intervals (panels 1 and 4). Scale bars: 10 µm (panels 1 and 4); 5 µm (panel 7). (B) COS-7 cells expressing the NPC1 (I1061T) mutant construct (panel 1) or subjected to shRNA and rescue experiments with wild-type (W.T.) NPC1 (panels 2 and 3) or with the NPC1 (I1061T) mutant (panels 4 and 5). The time-lapse sequence (from the boxed area) depicts 1 s intervals. Higher-magnification images of boxed areas from panels 2 and 4 highlight membrane morphology (panels 3 and 5). Scale bars: 10 µm (panel 1) or 5 µm (panels 2 and 4). Western blot analysis of cells transfected with non-targeting (lane 1) or NPC1-targeting (lane 2) shRNA using anti-tubulin as a loading control (panel 6). (C) Differential accumulation of SREBP1 in ER membranes or the nucleus. SREBP1 in branching ER membranes (yellow arrow) in wild-type COS-7 cells (panel 1). SREBP1 accumulation in NPC24 human biopsy cells expressing the homozygous mutant (I1061T) of NPC1 (panel 2). SREBP1 accumulation in COS-7 cells transfected with StARD9 shRNA (panel 3) and co-expressing eGFP as a transfection marker (inset). Scale bar: 10 µm. Images are representative of three independent experiments.

Although it is well-accepted that mutations in NPC1 are responsible for ∼95% of human cases of NPC disease (Carstea et al., 1997), there is less consensus on the specific cellular activities that NPC1 is responsible for. For example, both the N-terminal domain and the sterol-sensing domain of NPC1 are thought to bind cholesterol and mediate the movement of lumenal cholesterol across the limiting membrane of LE/Ls. However, the lipids that over-accumulate in NPC disease are not limited to cholesterol and include oxysterols, sphingolipids, gangliosides and GPI-anchored proteins. Furthermore, the pathological accumulations of cholesterol have been proposed to affect the functional state of LE/Ls directly, including perturbing Ca^2+^ uptake, autophagy and/or mitophagy, and the thickness of the glycocalyx. However, the specific ways in which NPC1 mutations affect these parameters remain unclear. With this wide range of unknowns in mind, we generated a fluorescent protein (FP)-tagged NPC1-expressing plasmid construct for expression in mammalian cells. As seen by others previously (Zhang et al., 2001; Ko et al., 2001), the FP-tagged protein encoded by this plasmid accumulated in LE/Ls that clustered near the cell center but also displayed periodic excursions to the cell periphery (Fig. 1A). Also overlapping with previous observations, LE/L membranes incorporating FP-tagged NPC1 projected a population of long, slender, dynamic membrane tubules that emerged from the surface of stationary LE/Ls and extended towards the cell periphery (Fig. 1A; Movie 1). Tubule projection required microtubules and was inhibited by drugs that interfere with microtubule polymerization and dynamics, such as taxol and nocodazole (Fig. S1A).

To explore the nature of these tubules further, we co-expressed markers of the LE/L membrane or lumenal space with NPC1. NPC1 and LAMP1 colocalized to the same tubules (Fig. 1A) indicating the presence of a LE/L limiting membrane. NPC1 also colocalized with the soluble LE/L enzyme cathepsin B (CTSB) (Fig. 1A; Movies 2–4), indicating the presence of a lumenal space within the tubules capable of delivering cholesterol or other lumenal biomolecules. These markers labeled both LE/Ls and tubulated projections when expressed alone or in combination (Fig. S1B). Tubulated LE/L membranes were also detected in fixed cells stained for LE/L proteins (Fig. S1C). This suggests that the novel structures incorporating NPC1 are hollow, membrane-bounded, tubular membranes.

Because the NPC1 and NPC2 proteins contain cholesterol-binding sites, we imaged 22-[*N*-(7-nitrobenz-2-oxa-1,3-diazol-4-yl)amino]-23,24-bisnor-5-cholen-3β-ol (NBD)-tagged cholesterol with NPC1. This imaging revealed the presence of cholesterol in the tubules, suggesting a potential role for these tubules in cholesterol movement out of LE/Ls (Fig. S1A). Consistent with this prediction, co-expression of markers for NPC1 and the endoplasmic reticulum (ER) suggested contact between LE/L tubules and the branching membranes of the ER (Fig. S1A). Taken together, these results identify membranous tubule projections as a novel mechanism of cholesterol export from LE/Ls that could be responsible for delivering endocytosed lipids to downstream membranous compartments for utilization.

The most common mutation in human NPC1 is I1061T and it is responsible for ∼20% of human NPC disease cases (Carstea et al., 1997). To compare wild-type and mutant NPC1 in these assays, we expressed a FP-tagged NPC1 (I1061T) construct and performed live-cell imaging. Strikingly, the mutant protein accumulated in LE/L membranes to an extent comparable to wild-type NPC1 (Fig. 1B). However, these membranes failed to project the dynamic membrane tubules observed when using the wild-type construct (see Movie 5). Because the COS-7 cells used for these assays express endogenous, wild-type NPC1, we performed shRNA coupled with rescue to deplete the native NPC1 protein and allow us to focus on the FP-tagged proteins (Fig. 1B). shRNA against native NPC1 and rescue with wild-type NPC1 mimicked the previous experiments and revealed projection of dynamic LE/L tubules (Fig. 1B). In contrast, shRNA against native NPC1 and rescue with NPC1 (I1061T) produced large spherical LE/Ls with a diameter suggesting engorged lumens (Fig. 1B). These mutant LE/Ls were less motile and failed to project any LE/L tubules. This suggests that LE/Ls incorporating mutant NPC1 are defective in tubule projection, potentially impairing the export of endocytosed cholesterol and other biomolecules from LE/Ls to other compartments.

Considering the possible destinations for LE/L cholesterol, we used the ER/nuclear localization of steroid response element-binding protein (SREBP1, also known as SREBF1) to assess LE/L-to-ER transport. Previous work by others revealed that SREBP1 is a cholesterol sensor that remains a transmembrane protein in the ER membrane if low-density lipoprotein (LDL)-derived cholesterol is transported successfully from LE/Ls to the ER (Wang et al., 1994). However, under conditions that block LE/L-to-ER transport of cholesterol, SREBP1 undergoes proteolytic cleavage and nuclear import to drive transcription of genes needed for *de novo* cholesterol biosynthesis. Therefore, the cellular location of SREBP1 can be used as an indicator of LE/L-to-ER cholesterol transport. SREBP1 imaging in cells expressing wild-type NPC1 (i.e. COS-7 cells) revealed the typical reticular ER localization pattern with some ER accumulation around the nuclear envelope (Fig. 1C). This indicates retention of SREBP1 in the ER and successful LE/L-to-ER transport of cholesterol. In contrast, cells homozygous for NPC1 (I1061T) displayed a strikingly different pattern. The reticular ER localization for SREBP1 was lost, and SREBP1 accumulated extensively in the nucleus (Fig. 1C). This indicates a lack of LE/L-to-ER transport, potentially because of a loss of LE/L tubulation.

Overall, these experiments suggest that NPC1 is a component of LE/L tubulation and that this activity is impaired by mutations in NPC1. Furthermore, the loss of LE/L tubulation reduces the transport of LDL-derived cholesterol from LE/Ls to the ER, potentially inducing the pathological accumulation of cholesterol and other lipids in LE/Ls that defines NPC disease.

### Identification of StARD9 as a LE/L kinesin

LE/L tubulation is recognized in many cell types, including macrophages in which tubulation contributes to the formation of phagocytic compartments and elimination of pathogens (Hollenbeck and Swanson, 1990). Several molecules are implicated in tubule formation (Gokool et al., 2007). However, current models for tubule formation do not fully-explain the behaviors we observed for NPC1-containing membranes, including vectorial, microtubule-dependent tubule projection and rapid redirection along linear elements. To identify candidates for the dynamic tubule projections we observed, we generated a stable cell line (COS-7) expressing FP-tagged wild-type NPC1. Intact (i.e. non-extracted) NPC1-containing membranes were purified by membrane flotation and immunoprecipitation (Fig. S2A) to complete a proteomic survey of the cytoplasmic face of these LE/Ls. Tryptic fragment data was used to identify candidates, focusing on proteins that shared some type of microtubule binding, some connection to cholesterol and some indication of LE/L localization.

**Fig. 2.**
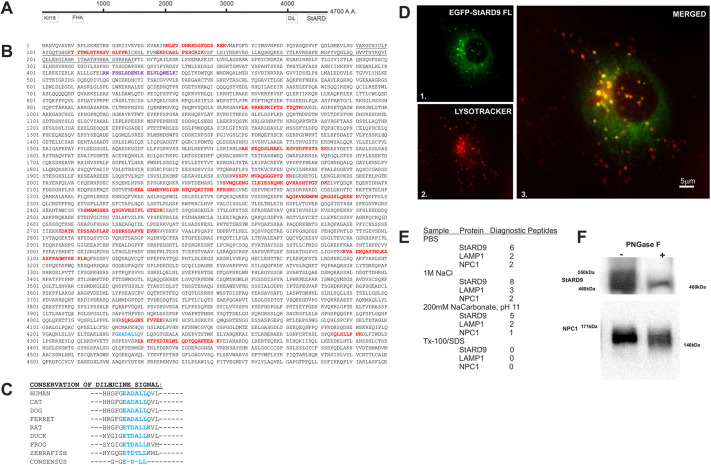
**Cloning of full-length StARD9.** (A) Line diagram depicting domains and features in StARD9. The locations of the KIF16 kinesin motor domain, forkhead-associated (FHA) domain, dileucine Signal (DiL) and steroidogenic acute regulatory domain (StARD) domain are indicated. (B) Deduced amino acid sequence of StARD9 cloned by RT-PCR. Sequences of tryptic peptides from StARD9 in isolated NPC1-containing membranes are indicated in red. The dileucine signal is indicated in blue. A 20 amino acid stretch (60 bp) annotated in GenBank was not present in our cloned sequences (purple). (C) Alignment of sequences surrounding the dileucine signal from representative vertebrate species. (D) Colocalization of full-length EGFP–StARD9 (panels 1 and 3) with LysoTracker (panels 2 and 3) in motile LE/L membranes. See also Movies 6–8. Scale bar: 5 µm. (E) Summary of MRM analysis of control, salt-extracted, carbonate-extracted and detergent-extracted phagosomes (see also Table S1). Diagnostic peptides from StARD9, LAMP1 and NPC1 are indicated as proteomic markers. Tx-100 indicates Triton X-100. (F) Mouse cerebellar extracts were treated without (−) or with (+) PNGase F to remove N-linked glycosylation and immunoblotted for StARD9 and NPC1. The StARD9 and NPC1 blots are from separate experiments. Images are representative of three independent experiments.

A number of microtubule motor proteins, including cytoplasmic dynein, were identified among the large list of candidates. Interestingly, one candidate emerged in the list that met each of our criteria. StARD9 was identified by 15 tryptic peptides spread across its predicted 4700 amino acid (aa) sequence suggested by *in silico* analysis (Fig. 2; Fig. S2B). Among the interesting features of this sequence are an N-terminal kinesin domain (KIF16) and a C-terminal cholesterol-binding domain (StARD) that defines the StART family (Fig. 2A). Although partial sequences for the N-terminus (Nakagawa et al., 1997; Torres et al., 2011) or C-terminus (Halama et al., 2006) for StARD9 have been cloned independently, full-length cDNA cloning efforts have lagged for StARD9, resulting in some confusion in the literature about the identity and cell biology of this protein. Furthermore, a previous StARD9 study reports an important role for StARD9 in mitotic spindle formation (Torres et al., 2011), which appears different from a role in LE/L function.

To resolve some of these important issues, we undertook the complete cloning of *STARD9* from human embryonic mRNA. Sequences encoding the C-terminal third of StARD9 were obtained from existing expressed sequence tag (EST) projects (Nagase et al., 2000), whereas sequences encoding the N-terminal and middle portions of the sequence were cloned by reverse-transcription PCR (RT-PCR). Using sequence overlap, we assembled the complete sequence encoding the ∼14 kb *STARD9* cDNA (Fig. 2A,B), producing a full-length StARD9 construct that could be used for subsequent analysis.

Our StARD9 sequence encodes each of the 15 tryptic peptides we identified originally and the signature motor domain sequence of KIF16B (Fig. S2C) used previously to define the kinesin family (Nakagawa et al., 1997). A 60 bp deletion in our sequence near the N-terminus differs from the *in silico* predictions in GenBank (Fig. 2B). We also identified a conserved dileucine (DiL) signal near the C-terminus (Braulke and Bonifacino, 2009) that is shared with NPC1 and other LAMPs as a LE/L-targeting signal (Fig. 2B,C). Taken together, these data confirm the N-terminal kinesin domain and C-terminal StART domain but also identify a C-terminal LE/L-targeting signal in StARD9. This suggests that StARD9 is a previously unknown LE/L kinesin, in addition to it having potential roles in mitotic spindle formation (Torres et al., 2011).

Using our constructs for full-length StARD9, we generated a new FP-tagged version for expression in mammalian cells. These expression experiments revealed prominent accumulation of StARD9 in membranes that were also positive for LysoTracker and displayed a typical bidirectional saltatory motion in live-cell imaging (Fig. 2D; Movies 6–8). This suggests that StARD9 is a LE/L microtubule motor protein that mirrors NPC1 localization during interphase. To assess where StARD9 accumulates during mitosis, we compared the expression of constructs encoding the motor domain only to that of the construct encoding full-length StARD9 (Fig. S2E). The motor domain-expressing construct labeled the mitotic spindle prominently, as reported previously (Torres et al., 2011). In contrast, full-length StARD9 labeled membranes that contained LAMP1 during early and late stages of mitosis.

To explore the potential mechanisms of LE/L accumulation, we performed proteomic tandem mass spectrometry (MS/MS) analysis of latex bead-loaded phagosomes subjected to different extractions. Latex bead-loaded phagosomes provide a biochemically pure population of NPC1 and LAMP1-containing membranes with significant advantages over other preparations (Blocker et al., 1997). These membranes were subjected to incubation with: (1) PBS, (2) 1 M NaCl, (3) 200 mM sodium carbonate (pH 11) or (4) 1% Triton X-100 with 1% SDS; followed by multiple reaction monitoring (MRM) analysis of tryptic fragments of LAMP1, NPC1 and StARD9. MRM analysis was used as a detection method because of its significant quantitative and dynamic range advantages over western blot analysis (Li et al., 2012). Similar to LAMP1 and NPC1, StARD9 was retained in membranes incubated with (1) PBS, (2) 1 M NaCl and (3) 200 mM sodium carbonate, but not in membranes extracted with Triton X-100/SDS (Fig. 2E; Table S1). StARD9 detection levels after Triton X-100/SDS extraction were more than 10,000-fold lower than those in the other three conditions, comparable to the results for NPC1 and LAMP1. This suggests a robust mechanism of membrane association for StARD9, potentially important for LE/L tubulation.

Proteins synthesized in the secretory pathway often display N-linked glycosylation, so another indication of membrane association is sensitivity to PNGase F digestion (Alvarez-Manilla et al., 2006). Using cerebellum tissue as a protein source, we compared control and PNGase F-digested samples by western blot analysis. NPC1 displayed a reduction in band complexity and an increase in gel mobility following PNGase F treatment, indicating N-linked glycosylation for normal samples (Fig. 2F). Although significantly larger than NPC1, StARD9 also displayed a reduction in band complexity and increased gel mobility following PNGase F treatment (Fig. 2F). Taken together, these findings suggest that StARD9 undergoes N-linked glycosylation, similar to NPC1 and other proteins with membrane association.

### Requirement for StARD9 in LE/L membrane motility

**Fig. 3.**
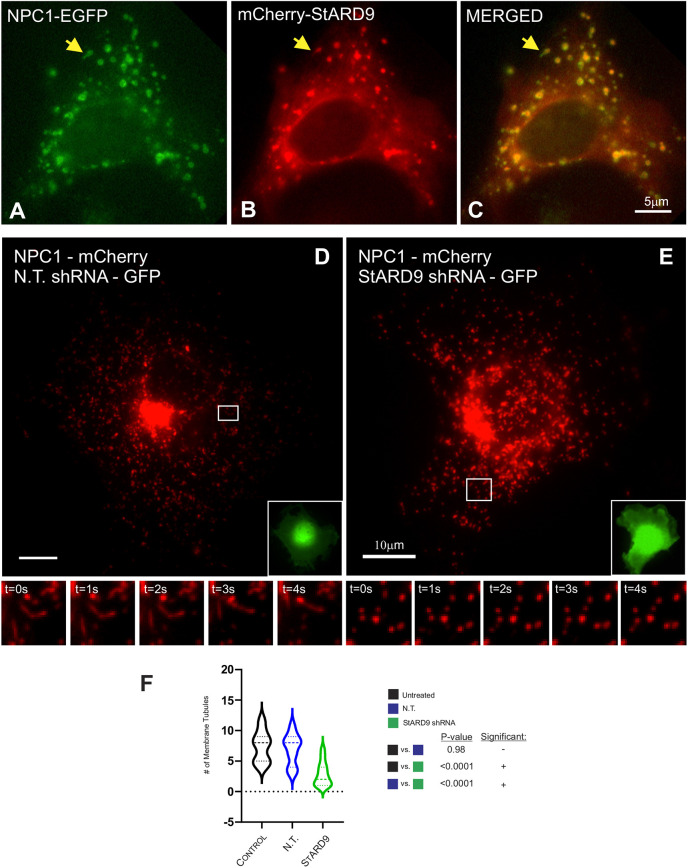
**Colocalization of StARD9 with NPC1 and the requirement for StARD9 in tubulation of NPC1-containing membranes.** (A–C) COS-7 cells expressing both NPC1–EGFP (A,C) and mCherry–StARD9 (B,C) display extensive colocalization in large spherical membranes that display tubulation (yellow arrows; see also Movies 9–11). Scale bar: 5 µm. (D,E) Tubulation of NPC1–mCherry-containing membranes in COS-7 cells expressing non-targeting (N.T.) shRNA (D) but not the StARD9-targeting shRNA (E). Scale bar: 10 µm. Insets indicate GFP marker of shRNA transfection. Time-lapse sequence images from boxed regions reflect 1 s intervals. (F) Violin plots of tubulation quantification from three separate transfections (*n*=15 cells each) reveals a significant loss of lysosomal tubulation in cells depleted of StARD9 (green violin) compared to control cells (black violin) or cells expressing the non-targeting (N.T.) shRNA (blue violin). Dashed line in violins represent median values and dotted lines indicate the 25th and 75th percentiles. *P*-values were obtained from two-tailed unpaired Student's *t*-test.

**Fig. 4.**
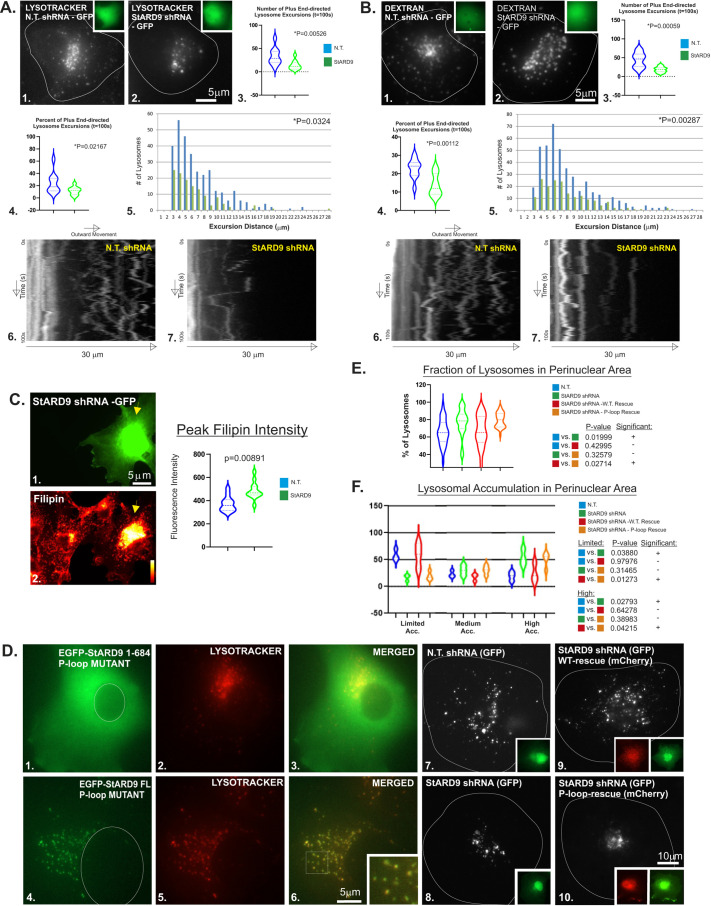
**Impact of StARD9 depletion on lysosome motility.** (A,B) Non-targeting (N.T.; panel 1) and StARD9-targeted (panel 2) shRNA constructs were transfected into COS-7 cells labeled with LysoTracker (A) or by pulse chase with fluorescent dextran (B). Cells are outlined in white. Insets show co-expression of GFP with the shRNA construct. Scale bar: 5 µm. The number of plus end-directed excursions (panel 3), the percentage of lysosomes undergoing plus end-directed motility (panel 4) and the run length of excursions (panel 5) in 100 s were compared. *P*-values were obtained from two-tailed unpaired Student's *t*-test. Kymograph analysis of lysosome motility highlights the reduction in centrifugal motility in StARD9-depleted cells (panels 6 and 7). (C) Measurements of peak filipin intensity to assess cholesterol accumulation in cells depleted of StARD9 (yellow arrows). Scale bar: 5 µm. (D) The P-loop mutant construct spanning aa 1–684 (panels 1–3) displays a soluble cytoplasmic localization pattern (panel 2). Full-length (FL) StARD9 harboring the P-loop mutation (panels 4 and 6) incorporates into LysoTracker-positive, non-motile LE/Ls (panel 5). White circles indicate the nucleus. The inset in panel 6 displays a higher-magnification image of the boxed area. See also Movie 12. Scale bar: 5 µm. The dispersed localization of dextran-labeled LE/Ls (white) reflects the typical bidirectional motility in cells treated with a non-targeting (N.T.) shRNA (panel 7) but not in cells depleted of StARD9 (panel 8). Lysosome motility was restored in cells depleted of StARD9 and rescued with a shRNA-resistant wild-type StARD9 construct (panel 9) but not restored in cells treated with StARD9 shRNA and rescued with a shRNA-resistant P-loop mutant construct (panel 10). Green (GFP) insets represent the shRNA plasmid reporter; red (mCherry) insets indicate the ‘rescue’ construct. Cells are outlined in white. Scale bar: 10 µm. (E) The percentage of LE/Ls accumulated in a 24 µm perinuclear circle as an indicator of transient plus end-directed excursions of dextran-labeled LE/Ls. Cells expressing the non-targeting (N.T.) shRNA (blue violin); cells depleted of StARD9 (green violin); cells depleted of StARD9 and rescued with a full-length shRNA-resistant wild-type StARD9 construct (red violin); and cells depleted of StARD9 and rescued with a full-length shRNA-resistant P-loop mutant construct (orange violin). *n*=3 (ten cells each). *P*-values were obtained from two-tailed unpaired Student's *t*-test. (F) The percentage of cells with limited, medium or high levels of perinuclear accumulation (Acc.) of lysosomes was calculated in cells treated with a non-targeting (N.T.) shRNA (blue violins); StARD9 shRNA (green violins); StARD9 shRNA and rescue with wild-type StARD9 (red violins); or StARD9 shRNA and rescue with a P-loop mutant of StARD9 (orange violins). *P*-values were obtained from two-tailed unpaired Student's *t*-test. *n*=3 (ten cells each). For violin plots, the dashed lines represent median values and dotted lines indicate the 25th and 75th percentiles.

Based on the finding that StARD9 is a novel LE/L kinesin responsible for LE/L tubulation, we considered several predictions for StARD9 activity. First, if StARD9 and NPC1 participate in the same tubulation process, they should reside in the same membranes. Taking advantage of our cloning of full-length StARD9, we co-expressed StARD9 and NPC1 for live-cell imaging. Consistent with our model, StARD9 and NPC1 accumulated in the same LE/L membranes and incorporated into the same LE/L tubules that projected from the LE/L surface (Fig. 3A–C; Movies 9–11). To test the requirement for StARD9 in tubulation, we used shRNA to deplete endogenous StARD9 (Fig. S3) and assessed LE/L tubulation of NPC1-containing membranes. StARD9 depletion reduced LE/L tubulation significantly (Fig. 3D–F), suggesting an important role for StARD9 in the projection of membrane tubules. However, when we assessed the overall motility of LE/Ls, we also observed a general impairment of LE/L motility, as indicated by kymograph analysis and quantitative analysis of individual membrane motility (Fig. 4A,B). Depletion of StARD9 reduced the number of membrane excursions towards the cell periphery (per 100 s), the percent of membranes than underwent excursions (per 100 s) and the run length of excursions using either LysoTracker or endocytosed fluorescent dextran as the membrane marker. Kymograph analysis suggested the immobilization of most LE/L membranes after StARD9 depletion (Fig. 4A,B). To assess relevance for cholesterol transport, we depleted COS-7 cells of StARD9 by shRNA and then measured the impact on cholesterol accumulation in LE/Ls (Fig. 4C). Similar to NPC1 mutations, StARD9 depletion induced a significant increase in peak cholesterol accumulation as indicated by filipin labeling (Fig. 4C). Finally, depletion of StARD9 by shRNA also drove an accumulation of SREBP1 in the nucleus, indicating a loss of LE/L-to-ER cholesterol transport (Fig. 1C). This suggests that StARD9 plays a significant role in LE/L behavior overall, including motility, tubulation and cholesterol trafficking.

Using the degree of perinuclear clustering as an indicator of excursions of LE/Ls to the cell periphery, we tested the requirement for StARD9 activity. Cells expressing the non-targeting shRNA displayed ∼65% perinuclear accumulation of lysosomes (Fig. 4D–F), whereas perinuclear accumulation of lysosomes increased to 77% in cells depleted of StARD9 (Fig. 4D–F). This suggests a requirement for StARD9 in the periodic excursions of LE/Ls to the cell periphery. Rescue of StARD9 shRNA depletion with a full-length shRNA-resistant wild-type StARD9 construct shifted the perinuclear accumulation back to ∼66% (Fig. 4D–F), reflecting a return to excursions into the cell periphery. We generated a P-loop mutation (Silvanovich et al., 2003) in StARD9 that impaired microtubule binding (see Fig. 4D–F; Fig. S4) to assess a requirement for microtubule-dependent motility by StARD9. Rescue of StARD9 shRNA depletion with a full-length shRNA-resistant P-loop mutant construct revealed substantial perinuclear accumulation (Fig. 4D–F; Movie 12) comparable to StARD9 depletion alone, suggesting a loss of excursions into the periphery. Taken together, these results suggest that StARD9 is required for motility from the perinuclear region to the cell periphery, in addition to a role in LE/L tubulation.

**Fig. 5.**
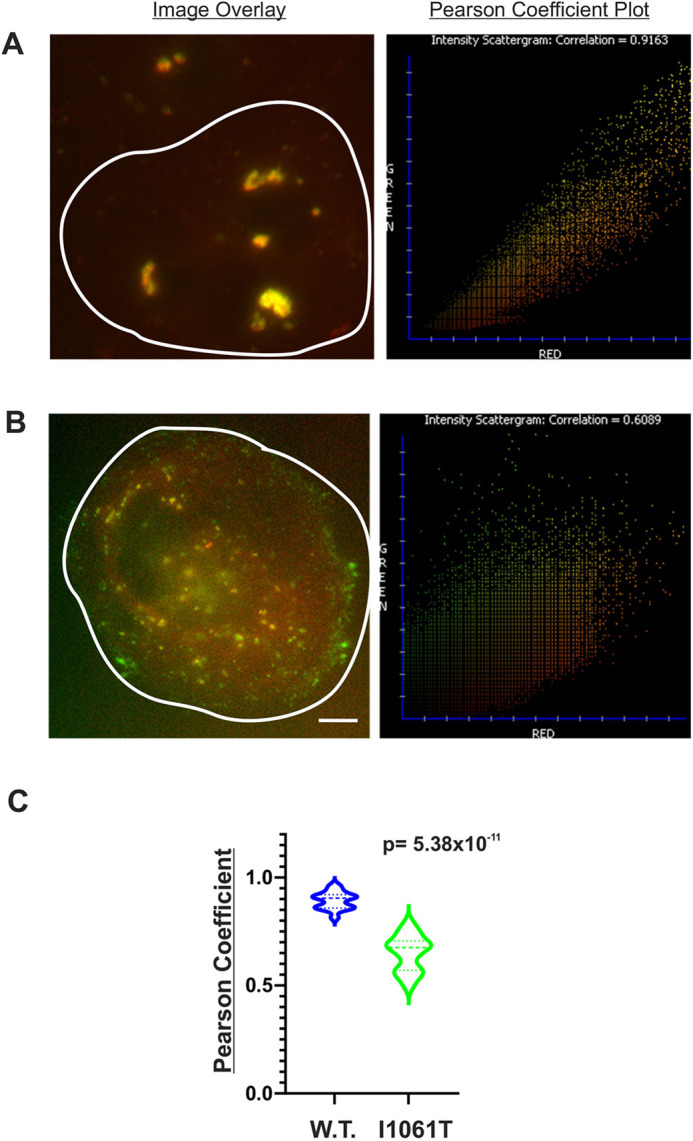
**Impact of NPC1 mutation on colocalization with StARD9.** (A,B) Degree of colocalization of mCherry–StARD9 (red) with wild-type NPC1–EGFP (A, green) or with I1061T mutant NPC1–EGFP (B, green), as indicated by the sample image (left) and calculated Pearson correlation coefficient (right). Cells are outlined in white. *n*=10. Scale bar: 5 μm. (C) Summary of comparison of cells analyzed (*n*=10 each) by Pearson coefficient test. Dashed lines in violins represent median values and dotted lines indicate the 25th and 75th percentiles. *P*-values were obtained from a two-tailed unpaired Student's *t*-test.

Because we observed a loss of LE/L tubulation in cells expressing mutant NPC1, we investigated how NPC1 mutations might affect StARD9 functionality. As an initial indicator of changes in LE/L proteomics, we generated stable cell lines expressing FP-tagged NPC1 (I1061T) and isolated LE/L membranes as described above for wild-type NPC1. Proteomic mapping studies of these membrane failed to identify StARD9, suggesting some difference in membrane protein composition (data not shown). Because wild-type StARD9 colocalized extensively with wild-type NPC1 (Fig. 3), we repeated the same analysis for the NPC1 (I1061T) mutant construct (Fig. 5A–C). Interestingly, we observed a significantly lower level of colocalization from ∼95% for wild-type NPC1) to ∼65% for NPC1 (I1061T) (Fig. 5A–C). This suggests that mutations in NPC1 potentially affect the generation of LE/Ls with both NPC1 and StARD9, thereby impairing the ability to project membrane tubules and mediate bidirectional motility.

### CRISPR/Cas9 removal of StARD9 induces NPC disease

A signature feature of NPC disease is the progressive loss of Purkinje cells in the cerebellum, which correlates with the onset and severity of neurodegeneration symptoms. To test whether StARD9 is required for the same Purkinje cell survival as NPC genes, we generated StARD9 KO mouse lines using CRISPR/Cas9 excision in one-cell mouse embryos. Ten mutant mouse lines were produced from 50 embryos (Fig. S5), indicating a significantly better success rate than previous methods of producing KO mice. Some of the new mouse lines shared the same sequences surrounding the guide RNA targets, rendering the tracking of specific lineages by genotyping impossible. As a result, we expanded lines with unique genotyping sequences for further analysis (Fig. S5).

**Fig. 6.**
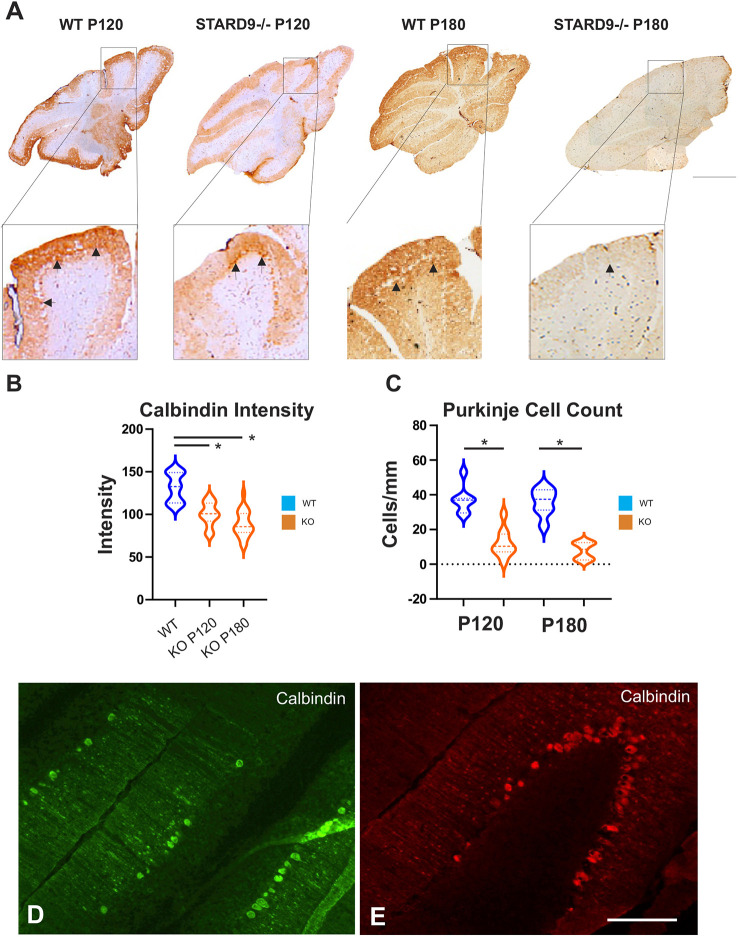
**StARD9 is required for Purkinje cell survival.** (A–E) Histological analysis of StARD9(−/−) mice. (A) Frozen sagittal sections of cerebella from wild-type (WT) and StARD9(−/−) mice at postnatal days P120 and P180 were stained for Purkinje cells using calbindin as a marker (black arrows). Scale bar: 100 mm. (B) The intensity of calbindin staining in the molecular layer was used as an indicator of Purkinje cell loss in StARD9 (−/−) (KO) mice (*n*=5). (C) Comparison of the number of Purkinje cells per mm in wild-type (WT) and StARD9 (−/−) (KO) mice at P120 and P180 (*n*=5). Dashed lines in violins represent median values and dotted lines indicate the 25th and 75th percentiles. *P<0.05; *P*-values were obtained from a two-tailed unpaired Student's *t*-test. (D,E) Calbindin staining of frozen sections from P120 StARD9 (−/−) mice as a marker of Purkinje cells in the lateral edges of each lobe (panel D) and the apex of each lobe (panel E). Scale bar: 10 mm.

StARD9 KO mice developed comparable neurodegeneration phenotypes to those of NPC mutant mice, with loss of Purkinje cells (Fig. 6A–E) and the development of tremors, ataxia, loss of grip strength and abnormal walking gait (Table S2). The onset of symptoms was closer to NPC1 (I1061T) mutant mice than NPC1(−/−) mice (Loftus et al., 1997; Praggastis et al., 2015). However, the pattern of phenotype presentation and the progression towards mortality was analogous (Davidson et al., 2009).

Consistent with our understanding of NPC disease, StARD9 KO mice displayed a progressive loss of Purkinje cells (Fig. 6A–E). Purkinje cell loss initiated in lobes 1–2 of the cerebellum but then advanced towards lobe 9 in a progressive pattern over time. Mimicking NPC disease, this correlated with the development of more severe symptoms of neurodegeneration (Table S2). Within individual lobes of the cerebellum, the loss of Purkinje cells appeared somewhat stochastic, with greater loss along the lateral edges than the apical tips (Fig. 6D,E). This pattern progressed over time towards the more distal lobes. Overall, loss of StARD9 mimicked the neurodegenerative features of NPC disease. This suggests that StARD9 is intimately involved in the process that NPC genes are responsible for.

## DISCUSSION

Mutations in NPC1 have been implicated in causing ∼95% of human Niemann–Pick type C disease cases. However, the mechanisms by which NPC1 mutations impair function remain controversial. A number of consequences of NPC mutations have been reported, including changes in LE/L Ca^2+^ content (Tiscione et al., 2019), ineffective cholesterol export to the plasma membrane (Kanerva et al., 2013) and a reduction in the thickness of the glycocalyx (Li et al., 2015). Comparing membranes incorporating wild-type versus I1061T mutant NPC1, we propose that a significant consequence of NPC1 mutations is loss of lysosomal tubulation. Motility differences in membranes containing NPC1 have been reported by others. However, Zhang et al. (2001) attribute these changes to excessive cholesterol and Ko et al. (2001) implicate overall motility of LE/Ls. An important advance in this study is the potential impact of NPC1 mutations on the projection of lysosomal tubules from the surface of LE/Ls. Because it appears that LE/L tubulation provides a vectorial and deliberate mechanism of intracellular cholesterol delivery, loss of tubulation could explain both the pathological accumulation of cholesterol in NPC disease LE/Ls and the apparent inability to deliver cholesterol to the ER for proper sensing. It could also explain the aberrant mTORC1 signaling detected in NPC disease (Lim et al., 2019). Loss of LE/L tubulation could also provide the basis for pathological accumulation of the many lipids beyond cholesterol (oxysterols, sphingolipids, gangliosides and GPI-anchored proteins) that typify NPC disease.

Neither NPC1 nor NPC2 contain the features one might expect for LE/L tubulation. A search for additional components of this pathway identified StARD9 as a previously unknown LE/L kinesin. StARD9 contains a DiL signal, a signature feature of LAMPs that is shared with NPC1. Expression of full-length StARD9 revealed accumulation in LE/Ls. Furthermore, shRNA-based depletion of StARD9 reduced LE/L tubulation to an extent comparable to that seen for NPC1 mutants. Interestingly, analysis of StARD9 in the Human Protein Atlas project reveals prominent expression in Purkinje cells, which suggests lysosomal accumulation (Uhlen et al., 2010). Given the extensive work on other motor proteins implicated in LE/L function (Brown et al., 2005), it is surprising that StARD9 was not identified as a candidate previously. Human StARD9 is most similar to members of the KIF16 family (mouse nomenclature). However, the identities of members of the KIF16 protein family were suggested before cloning of these genes was complete. A comparison of the motor domain of StARD9 to these proteins reveals 93.7% conservation with KIF16B but 63.3% conservation with KIF16A (Fig. S2C). This suggests that StARD9 corresponds to KIF16B rather than KIF16A (Nakagawa et al., 1997). Interestingly, previous northern blot analysis suggested that *KIF16B* probes labeled a very large mRNA (Nakagawa et al., 1997), comparable to the StARD9 mRNA (∼16 kb). In contrast, *KIF16A* probes labeled a ∼5.2 kb mRNA, suggesting a more conventionally sized kinesin (Fig. S2D). Furthermore, the *STARD9* gene in humans is located on chromosome 15q15.2 encoding a 4700 aa protein with 36 exons. This locus is syntenic to *Kif16b* mouse chromosome 2.2EF, which encodes a 4585 aa protein containing 39 exons (Fig. S2D). In contrast, the human *KIF16A* gene is on chromosome 20p12.1 and encodes a 1317 aa protein with 29 exons. This is syntenic to mouse *Kif16a*, which is on chromosome 2.2G1 and encodes a 1312 aa protein with 29 exons (Fig. S2D). These and other aspects of StARD9 database annotations could have contributed to the absence of StARD9 in proteomic and other screens of LE/L motor proteins.

Unlike other members of the kinesin family, StARD9 displayed a robust association with LE/L membranes. Similar to LAMPs, StARD9 contains a conserved DiL signal near the C-terminus (Fig. 2B,C). This consensus element is thought to drive retrieval of LAMPs from the plasma membrane for delivery to LE/Ls (Braulke and Bonifacino, 2009). Differential extraction experiments revealed that StARD9 can be removed from membranes by detergent, but not by high salt or alkaline pH. This suggests that StARD9 is not a peripheral membrane protein. Finally, western blot analysis of StARD9 after PNGase F digestion suggested that StARD9 is subject to N-linked glycosylation, similar to NPC1. This feature suggests synthesis in the secretory pathway and exposure to the lumenal side of membranes. With a kinesin domain at the N-terminus, which is presumably on the cytoplasmic side of LE/L membranes, and N-linked glycosylation elsewhere in the molecule, StARD9 mimics bona fide membrane proteins. However, a complete topology map of StARD9 will be required to establish this feature.

Previous work suggests that StARD9 plays a role in spindle pole assembly during mitosis (Torres et al., 2011). Although this appears quite different from a role in LE/L motility, our studies do not eliminate its possible role in mitotic spindle function. Perhaps the post-mitotic nature of neurons emphasizes the LE/L function for StARD9 over mitotic functions.

We propose that LE/L tubulation could provide a mechanism to deliver LE/L-associated cholesterol to downstream membranes such as the ER and mitochondria. Tubulated membranes are already described for ER–Golgi transport, trans-Golgi network membrane transport and endocytic membranes (Lippincott-Schwartz et al., 1991; Watson and Stephens, 2005). A feature of tubulated membranes is the combination of the lipid bilayer and lumenal space that can contact and exchange with downstream membranes. The lipids known to be over-accumulated in NPC disease include cholesterol, oxysterols, sphingolipids, gangliosides and GPI-anchored proteins. LE/L tubules have the potential to drive the efflux of all of these lipids from LE/Ls. Furthermore, the loss of tubulation caused by NPC mutants could explain the complexity of over-accumulated lipids in LE/Ls, despite the presence of binding motifs for cholesterol but not for other lipids in NPC1 and NPC2. In other words, LE/L tubulation could be the more global function for NPC genes that is impaired in NPC disease.

Loss of Purkinje cells is a signature feature of NPC disease. Our newly generated StARD9 KO mice display a similar progressive loss of Purkinje cells that correlates with the onset and severity of neurodegeneration symptoms. Also similar to mouse models of NPC disease, StARD9 KO mice develop tremors, ataxia, loss of grip strength and abnormal walking gait in that order. Interestingly, a human patient with mutations in StARD9 was reported recently (Okamoto et al., 2017). Many of the symptoms in this patient resemble late-stage NPC disease, including neurodegeneration defects and epilepsy. This further supports the potential overlap in function between StARD9 and NPC genes. Hopefully, this study draws attention to the potential role of StARD9 mutations in neurodegeneration.

**Fig. 7.**
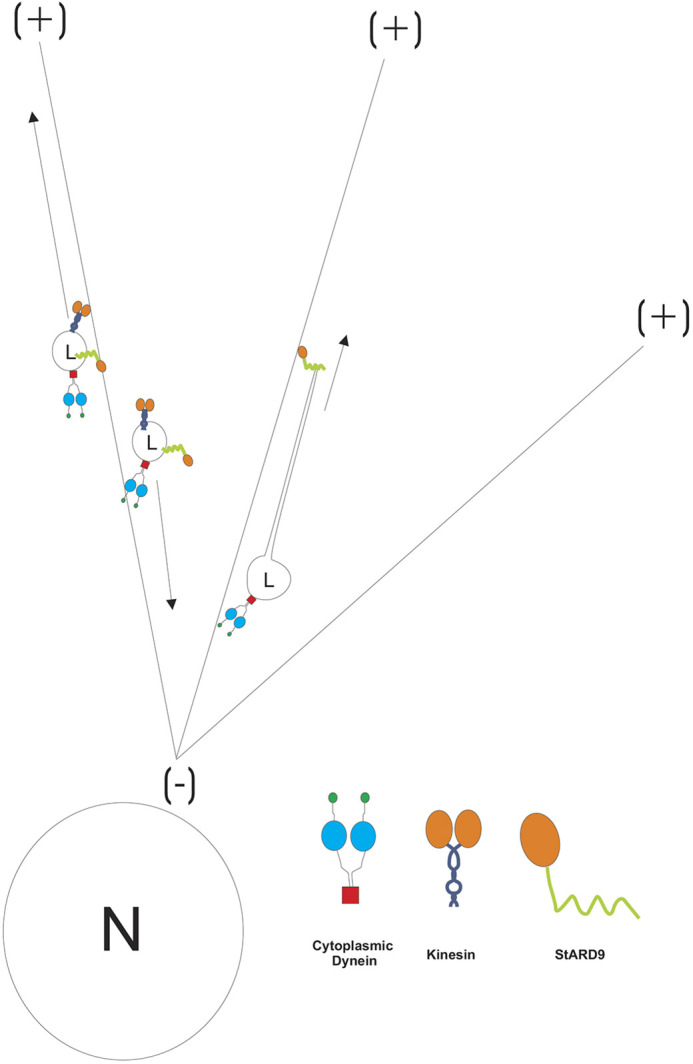
**Model for StARD9 function.** StARD9 depletion assays and StARD9 shRNA and rescue experiments suggest that StARD9 plays an important role in both the centrifugal excursions of LE/Ls (L) and the projection of membrane tubules. The monomer/dimer status of StARD9 is not known. However, the predicted presence of coiled-coil domains in StARD9 suggests dimerization. Based on previous work, cytoplasmic dynein is thought to function antagonistically through centripetal LE/L motility and anchoring of LE/Ls during projection of tubules. The contributions of dynactin and cargo adapter proteins for dynein are omitted for simplicity. Nucleus, N; lysosome, L; typical microtubule plus end, (+); typical microtubule minus end, (−).

In summary, StARD9 is a novel LE/L kinesin that contributes to both bidirectional motility of LE/Ls and projection of plus end-directed membrane tubules from the surface of LE/Ls (Fig. 7). Because mutations in NPC1 impact both these activities, we propose that NPC1 functions with StARD9 to regulate LE/L membrane dynamics. Loss of this regulation has the potential to explain the defects in LE/L cholesterol transport in NPC disease.

## MATERIALS AND METHODS

### Experimental models and subject details

#### Reagents

Chemical and standard reagents including filipin and NBD-cholesterol were obtained from Sigma-Aldrich, St. Louis, MO. LysoTracker and fluorescent dextran were obtained from Life Technologies (Grand Island, NY). shRNA constructs were obtained from SA Biosciences (Valencia, CA). Antibodies against tubulin [1:100 for immunofluorescence (IF), T6199, Sigma-Aldrich], EGFP (1:100 for IF, 632380, Clontech) and mCherry (1:100 for IF, NBP1-96752, Novus Biologicals) have been described previously (Whyte et al., 2008; Towns et al., 2009). Antibodies against StARD9 [1:100 for western blotting (WB), HPA014562] were obtained from Sigma-Aldrich. Site-directed mutagenesis utilized the Phusion kit (New England Biolabs, Ipswich, MA). Antibodies against NPC1 (5 μg/ml for IF, 1 μg/ml for WB, ab108921), LAMP1 (1:10 for IF, ab25630), SREBP1 (1:100 for IF, ab28481) and cathepsin B (1 μg/ml for IF, ab6313) were obtained from Abcam (Boston, MA). Filipin (f9765) was obtained from Sigma-Aldrich. NBD-cholesterol (N1148) was obtained from Invitrogen (Waltham, MA). BacMAM-ER (C10590) was obtained from Thermo Fisher Scientific (Waltham, MA). Nocodazole (M1404) and taxol (T7402) were obtained from Sigma-Aldrich. The sequences of oligonucleotides used in this study are provided in Table S3.

#### Plasmid constructs

The original NPC1 construct was obtained from Dr Ta Yuan Chang (Dartmouth University) and subcloned into pCI-neo (Promega, Madison, WI; E1841), placing the EGFP sequence at the C-terminus using standard methods. A similar approach was used to tag the C-terminus of NPC1 with mCherry. The NPC1 (I1061T) mutant construct was generated by PCR-based mutagenesis. shRNA constructs co-expressing GFP as a transfection reporter were obtained from SA Biosciences. The shRNA #276 (SA Biosciences, KH13606G) anneals to the StARD9 sequence at base pair 8138 and was identified as the most potent in StARD9 depletion. An EST clone encoding the C-terminal ∼1800 aa of StARD9 (KIAA1300) was obtained from Kazusa DNA Research Institute, Chiba, Japan (Nagase et al., 2000). The remaining portions of StARD9 were cloned by RT-PCR from human cerebellum RNA (Agilent Technologies, Wilmington, DE) and human liver mRNA (Life Technologies). Expression constructs for LAMP1 and cathepsin B were obtained from Michael Overholzer (Memorial Sloan Kettering Cancer Center).

#### Cell lines

Fixed and live-cell imaging was performed on COS-7 cells grown in Dulbecco's modified Eagle's medium (DMEM, Sigma-Aldrich) supplemented with 10% fetal bovine serum (Sigma-Aldrich), 1% L-glutamine and penicillin/streptomycin (Sigma-Aldrich). Transfections were performed using either Lipofectamine (Life Technologies), X-treme Gene DNA Transfection reagent (Roche, Indianapolis, IN) or NanoFect transfection reagent (ALSTEM, Richmond, CA). J774A.1 mouse macrophages were provided by Dr Jeff Schorey (University of Notre Dame) and cultured in the same medium. NPC24 cells were provided by Dr Forbes Denny Porter (National Institutes of Health) and were obtained with informed consent. The NPC1 (1061T/I1061T) genotype is confirmed, but no patient identifier information is known to the authors. The University of Notre Dame Institutional Biosafety Committee approved work with human cell lines. Cells were tested for contamination using PCR-based test kits (Sartorius, SMB95-1001).

#### LC-MS/MS analysis

Purified NPC1–EGFP-containing membranes were subjected to reduction and alkylation, digestion with trypsin or endoproteinase GluC (New England Biolabs) similar to that described previously (Champion et al., 2012; Williams et al., 2017). Samples were desalted by ZipTips (Millipore, Billerica, MA) according to the manufacturer's instructions and separated on a nanoAcquity UHPLC system (Waters Billerica, MA) prior to MS/MS analysis. Liquid chromatography (LC)/MS-MS analysis was performed on an amaZon ion trap instrument (Bruker Daltronics, Billerica, MA) running in data-dependent acquisition mode. Acquisition and processing were performed in the Mass Spectrometry and Proteomics Facility (MSPF) at the University of Notre Dame. Spectra were converted to .mgf files (MS/MS peak lists) using Data Analysis (Bruker) and subjected to database search with Mascot against the human UniProt database (downloaded 15 October 2009). MS tolerances were set to ±1.5 Da for MS1 and 1.7 Da for MS2. Owing to the small search space, traditional false discovery rate was not employed as it is not accurate for small-*n* datasets. A Mascot score of 40 and two peptides uniquely matching were used for ID cutoff.

#### LC-MRM analysis

LC-MRM data and transitions were selected and acquired in a manner nearly identical to that described previously (Llarrull et al., 2011; Li et al., 2012; Champion et al., 2009). MRM transitions were selected and monitored on a 5500 QTrap coupled with an Eksigent 2D NanoUHPLC (AB Sciex) running chromatography and gradient as described in the references above. Three or four transitions (as indicated) were selected for each peptide, and six peptides were selected for monitoring for StARD9, two peptides for LAMP1, and two proteotypic peptides were observed for NPC1. Peak integration and mapping were performed in Skyline (Pino et al., 2017).

#### Isolation of NPC1-containing membranes for MS/MS analysis

A stable cell line in COS-7 cells was generated using the pCI-neo (NPC1–EGFP) construct described above by standard methods. This cell line was expanded for biochemical isolation of NPC1-containing membranes. The cell pellet from five 150 cm tissue culture plates was subjected to hypotonic/mechanical lysis and brought to 2 M sucrose for separation in a 0.5 M/1.3 M/2 M sucrose gradient. The resulting membrane fraction at the 0.5 M/1.3 M interface was further subjected to immunoprecipitation with anti-NPC1 antibodies to isolate the NPC1-containing membrane fraction. This fraction was subjected to tryptic digestion without membrane solubilization to focus on the cytoplasmic face of the membranes. A protein profile was obtained using Mascot analysis of MS/MS results.

#### Preparation of purified phagosomes

J774A.1 mouse macrophages were fed fish-gelatin-coated 0.8 mm latex beads (Sigma-Aldrich) and allowed to internalize beads overnight. Treated cultures were subjected to mechanical lysis with a motorized pestle, brought to 2 M sucrose and loaded under a 0.5 M/1.3 M sucrose step gradient. After centrifugation at 30,000 ***g*** in a SW50 swinging-bucket rotor (Beckman Coulter), the phagosomes at the 0.5 M/1.3 M interface were isolated and for biochemical extraction. Extracted samples were processed for MS/MS analysis after solubilization by standard methods.

#### PNGase F analysis

Protein samples were digested from mouse cerebella or PANC-1 cells with RIPA buffer (150 mM NaCl, 1% NP-40, 0.5% sodium deoxycholate, 0.1% SDS and 50 mM Tris, pH 7.4) and mechanical homogenization. Samples were incubated with 5% SDS and 1 M dithiothreitol for 5 min at 95°C, then for 5 min at room temperature. Then, the samples were incubated with 1× PBS, 10% NP-40 and PNGase F (New England Biolabs) for 3 h at 37°C. The control reactions did not contain PNGase F. The samples were then subjected to immunoblotting. Multiple exposures of the StARD9 blot are presented in Fig. S6. The blot for NPC1 (Fig. 2F) is included for comparison and is from a different experiment from that of the StARD9 blot.

#### Time-lapse imaging

Microscopy experiments were performed on a Zeiss Axiovert 200M (Thornwood, NY) run by Metamorph software (Sunnyvale, CA). Fixed and live-cell imaging data were collected using either a Photometrics Coolsnap HQ or Cascade 1k CCD camera (Tucson, AZ). Time-lapse imaging of cells expressing NPC1–EGFP and mCherry–StARD9 utilized a dual-view camera (Photometrics) adapter to allow simultaneous acquisition of green and red channels at each timepoint. Kymographs were generated in Metamorph using a 1 µm slice from each frame.

#### Generation of StARD9(−/−) mice

CRISPR/Cas9 reagents were obtained from Transposagen (Lexington, KY) and provided to the Transgenic Core Facility (Purdue University) for injection into one-cell C57BL/6 mouse embryos. The resulting mice were genotyped by PCR to detect deletions of the *Stard9* gene. The resulting PCR products were cloned into plasmids and subjected to sequencing to define the precise junctions around the guide RNA sequence annealing sites. StARD9(+/−) mice were expanded for breeding to produce StARD9 (+/+), (+/−) and (−/−) mice in compliance with the Institutional Animal Care and Use Committee (IACUC) at the University of Notre Dame.

#### Mouse husbandry

Mice were housed socially in temperature-controlled room with a 12 h/12 h light-dark cycle. They were fed a chow diet. All caretaking and experiments were approved by the IACUC. Mice were euthanized between 2 and 6 months, or if they weighed 30% less than their littermates. Tissue from these mice was used for histology studies.

#### StARD9(−/−) mouse behavioral analysis

Mice were binned into three categories labeled 1–3. Category 1 corresponded to the least severe, category 2 as moderate and category 3 as severe. A composite score for symptom severity was developed from two measurements: coat hanger test and tremors. For the coat hanger test, mice were placed at the center of a standard coat hanger (3 mm diameter) by their front paws and observed for 30 s. Following observation, the mice were scored as follows: 1, remained on the coat hanger for at least 30 s; 2, fell off the coat hanger between 10 and 30 s; and 3, fell off the coat hanger in less than 10 s. The second observation was tremors. Mice were observed for 2 min by an individual who did not know the genotype and were scored based on the following: 1, no tremors; 2, minor tremors lasting for less than 10 s; and 3, major tremors lasting for more than 10 s. The two scores were averaged and are displayed in Table S2.

#### Cerebellum calbindin staining

Immunohistochemistry was performed as previously described (Praggastis et al., 2015). Cerebella were dissected and placed in 4% paraformaldehyde for 24 h at 4°C. After fixing, the tissue was placed in 20% sucrose at 4°C for 12 h. After being set in VWR Premium Frozen Section Compound (VWR, 95-57-838), 11 µm sections were made using a Leica CM1850 Cryostat and placed on VWR Superfrost Plus Slides (VWR, 48311-703). Sections were dried at room temperature for 30 min before staining. They were washed twice for 10 min in 1× PBS at room temperature. Sections were incubated in blocking solution (PBS with 1% bovine serum albumin, 1.5% donkey serum and 0.02% saponin) for 2.5 h at room temperature. Then, the sections were incubated overnight at 4°C in primary solution (1:3000 calbindin, Sigma-Aldrich, C9848). On the second day, the sections were washed four times in wash solution (PBS with 0.01% saponin) for 10 min. Then, the sections were incubated for 1 h at room temperature with biotinylated secondary antibodies (1:250 biotinylated goat anti-mouse IgG, Vector Laboratories, BA-9200). The sections were then washed five times for 10 min in wash solution, followed by incubation in VECTASTAIN ABC kit (Vector laboratories, PK-4000) for 1 h at room temperature. The sections were then washed four times for ten minutes in 1× PBS, followed by incubation in DAB Peroxidase Substrate Kit (Vector Laboratories, SK-4100) for 10 min. They were then rinsed twice with 1× PBS and mounted with Prolong Gold (Invitrogen, F9765) and stored at 4°C until imaging. Imaging was performed using a Leica S APO microscope. Color intensity was measured using ImageJ.

### Quantification and statistical analysis

#### Cultured cell experiments

The variable being measured, the number of independent experiments and the number of cells from which data were collected are specified in the legend for each figure. Data are presented as violin plots generated in GraphPad Prism. Statistical significance was determined using either two-tailed unpaired Student's *t*-test or one-way ANOVA with Brown–Forsythe post hoc test, depending on the experimental design.

#### StARD9(−/−) mouse experiments

Three mice from per genotype were analyzed per experiment. Images were analyzed using ImageJ for cell count and intensity. Means were calculated using Microsoft Excel and are presented as mean±s.e.m. A two-tailed unpaired Student's *t*-test was used to compare cell count and mean intensity.

### Sequences

The sequence accession code for all StARD9 Sequences is NM_020759.

## Supplementary Material

10.1242/joces.260662_sup1 Supplementary information
