# Supplementary material for: StARD9 is a novel lysosomal kinesin required for membrane tubulation, cholesterol transport and Purkinje cell survival
Source: J Cell Sci. 2023 Mar 2;136(5):jcs260662. doi: 10.1242/jcs.260662 (PMC10203878; doi:10.1242/jcs.260662)
Supplement: Supplementary information [file joces-136-260662-s1.pdf]

Supplemental Figure 1.

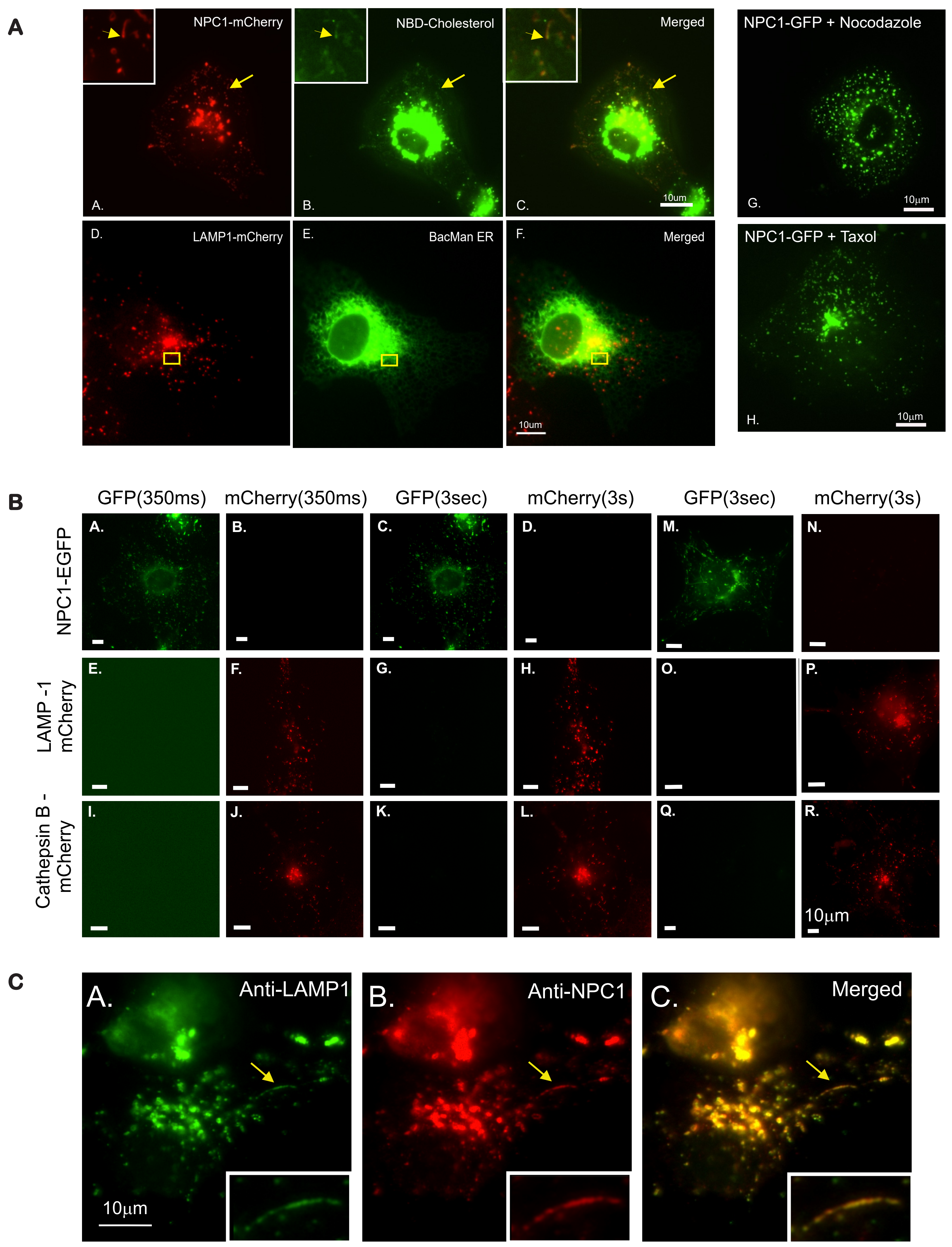

Journal of Cell Science • Supplementary information

Fig. S1. Imaging and Potential Functions for LE/L Tubulation. **A)** Incorporation of NBD- Cholesterol into LE/L Tubules. COS-7 cells expressing NPC1-mCherry (panels A&C) were provided NBD-cholesterol for live-cell uptake and labeling assays (panels B&C). NBD-cholesterol labeled individual NPC1 tubules (yellow arrows and insets). Mag. bar = 10 μm. Contact between LE/L tubules and the ER. Using LAMP- 1 as a marker of LE/L tubules (panels D&F), direct contacts between the LE/L tubules and ER membranes (BacMan ER) is observed by live-cell imaging (panels E&F). Mag. bar = 10 μm. LE/Ls labeled with NPC1-EGFP after incubation with nocodazole (panel G) or taxol (panel H). Mag. bar = 10μm. **B)** Single-channel imaging of COS-7 cells transfected with NPC1-EGFP (panels A-D, M,N), LAMP-1-mCherry (panels E-H, O,P) or Cathepsin B-mCherry (panels I-L, Q,R) after normal exposures (panels A,B,E,F,I,J) or excessive exposures (panels C,D,G,H,K,L) confirm a lack of signal bleed-through. Examples of cells transfected as above that display tubulation (panels M-R) also fail to display signal bleed-through during excessive exposures. Mag. bars = 10 μm. **C)** LE/L tubulation in fixed samples. COS-7 cells fixed and stained for LAMP-1 (panels A&C) and NPC1 (panels B&C) reveal LE/L tubulation. Mag. bar = 10μm.

Journal of Cell Science • Supplementary information

Supplemental Figure 2.

A.

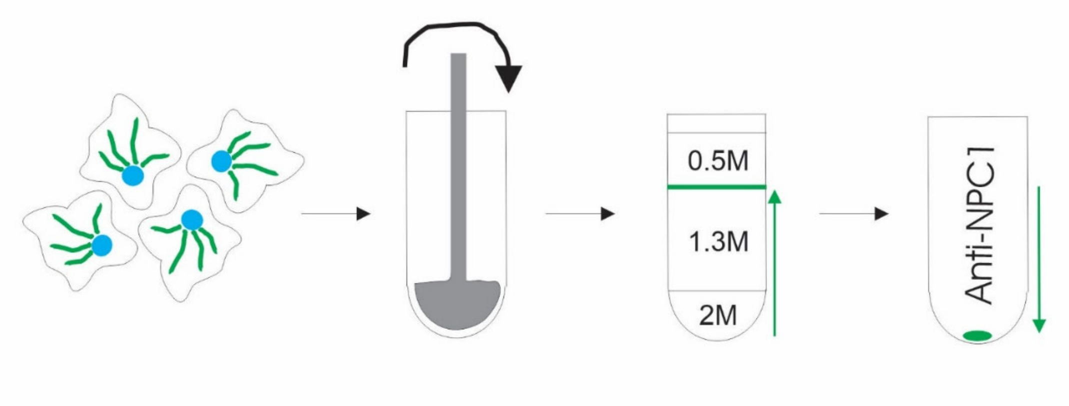

B.

Start - End	Observed	Mr(expt)	Mr(calc)	Delta	Miss	Sequence
37 - 53	1003.5000	2004.9854	2004.9501	0.0354	2	**R.NLKVDNRPDGFGDSREK.V** Carbamidomethyl (N-term); 2 Deamidated (NQ) (Ions score 17)
110 - 125	589.8000	1766.3781	1764.9080	1.4701	0	**K.TYTMLGTPVSVGLTPR.I** Carbamidomethyl (N-term); Oxidation (M) (Ions score 24)
134 - 147	854.2000	1706.3855	1706.8080	-0.4225	2	**R.EKDCASLPSSCRIK.V** Carbamidomethyl (N-term) (Ions score 21)
911 - 927	1050.8000	2099.5855	2097.1331	2.4525	2	**K.LKPRHEPKIFTSTTQTR.G** Carbamidomethyl (N-term); Deamidated (NQ) (Ions score 12)
1473 - 1496	906.4000	2716.1782	2715.2875	0.8908	2	**R.AEKEQDSLNAKLEGVSDFFSTSEK.E** Carbamidomethyl (N-term) (Ions score 12)
1870 - 1885	793.0000	1583.9854	1583.7978	0.1877	0	**R.VSSPVMVAQGGGPTPK.W** Carbamidomethyl (N-term); Oxidation (M) (Ions score 14)
1967 - 1996	1131.7000	3392.0780	3390.6837	1.3943	2	**R.SVMQLENGILEIESKQNKQVHASHTPGTDK.E** Carbamidomethyl(N-term);Oxidation (M)(Ions score 22)
2041 - 2069	1067.1000	3198.2781	3196.5207	1.7574	2	**R.DSEAGAMEVNSIGNHPQVQKITPNPFRSR.E** 2 Deamidated (NQ); Oxidation (M) (Ions score 26)
2185 - 2205	1174.9000	2347.7855	2347.0784	0.7071	2	**K.AQGKVEEMPMQRGGSLQEENK.V** 2 Deamidated (NQ) (Ions score 41)
2185 - 2205	1181.9000	2361.7855	2361.1053	0.6802	2	**K.AQGKVEEMPMQRGGSLQEENK.V** Oxidation (M) (Ions score 54)
2327 - 2349	802.7000	2405.0782	2404.0635	1.0147	0	**R.SMAMGSHSQSGVPESIPLGTEDR.I** 2 Oxidation (M) (Ions score 24)
2621 - 2647	937.2000	2808.5782	2807.3686	1.2096	2	**K.DATRTPSSADPLAPDSPRSSAPVEEVR.R** (Ions score 23)
3002 - 3027	943.6000	2827.7781	2825.3905	2.3876	2	**K.KVAEKQASTELEAASFPAGMYSEPLR.Q** Oxidation (M) (Ions score 23)
3938 - 3949	455.3000	1362.8781	1361.6939	1.1842	1	**R.SQRLGNSFVPEK.V** Deamidated (NQ) (Ions score 29)
4199 - 4206	970.9000	969.8927	969.5495	0.3433	0	**K.QLSLLPNK.D** Carbamidomethyl (N-term); Deamidated (NQ) (Ions score 10)
4243 - 4265	906.2000	2715.5782	2714.2129	1.3653	0	**R.SAHTPSDIELMLQDYQQAHEEAK.V** Carbamidomethyl(N-term;Deamidated(NQ);Oxidation(M(Ions Sc. 33)

C.

```
                              KIF16B: 91.4 % Identical (*) + 2.3 % Conservative Substitutions (:).
       :********************************:** **** * *  ******************* * ****************************:******* ********************
KIF16B-IFAYGQTGSGKTYTMLGTPASVGLTPRICEGLFIREDDCASRPCSRSIKVSFLEIYNERVRDLLKQSKQNKSYTLRVREHPEMGPYVQGLSQHVVTNYHQVIQLLEAGIANRITAATHVHEASSRSHA
StARD9-LFAYGQTGSGKTYTMLGTPASVGLTPRICEGLFVREKDCASLPSSCRIKVSFLEIYNERVRDLLKQSGQKKSYTLRVREHPEMGPYVQGLSQHVVTNYKQVIQLLEEGIANRITAATHVHEASSRSHA
KIF16A-IFTYGQTGSGKSYTMMGNSGDSGLIPRICEALFSRINETTRWDEASFRTEVSYLEIYNERVRDLLRRKSSKTFNLRVREHPKEGPYVEDLSKHLVQNYSDVEELMDAGNINRTTAATGMNDVSSRSHA
       :*:********:*** *      ** ***** ** *  : :          ** ************     *:: *******  ****  ** *:* **  *  *:: *  ** ****      ******
                              KIF16A: 55.5 % Identical (*) + 7.8 % Conservative Substitutions (:).
```

D.

Gene Name:	Human				Mouse			
	Chromosome Locus	Exons	mRNA	Amino Acids	Chromosome Locus	Exons	mRNA	Amino Acids
StARD9/KIF16B*	15q15.2	36	~16kb**	4700	2.2EF	39	~16kb***	4585
KIF16A*	20p12.1	29	~5.2kb**	1317	2.2G1	29	~5.2kb***	1312

Journal of Cell Science • Supplementary information

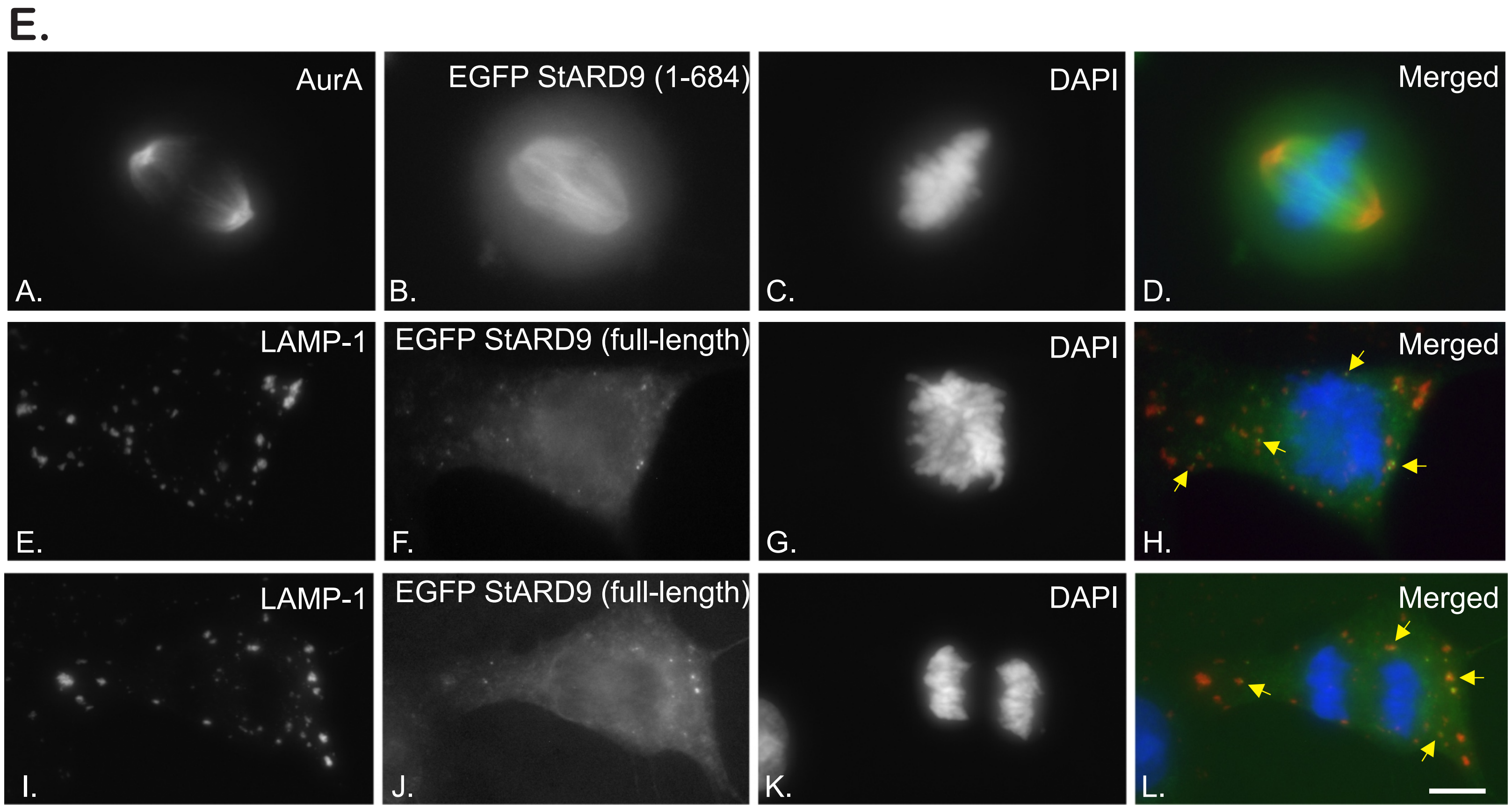

Fig. S2. Identification of StARD9 by MS/MS Analysis of LE/L Membranes. A) Membranes were purified from COS-7 cells stably-expressing wild-type NPC1 that were subjected to mechanical lysis in the absence of detergent followed by sucrose gradient flotation and immunoprecipitation with anti-NPC1 antibody. **B)** StARD9 was identified using 15 tryptic peptides. **C)** Human STARD9 was aligned with deduced amino acid sequences of RT-PCR-cloned mouse members of the KIF16 family (Nakagawa et al., 1997). StARD9 shares55.5% identity (ID) and 7.8% conservative substitutions (C.S.) with KIF 16A, but 91.4% identity (ID) and 2.3% conservative substitutions (C.S.) with KIF 16B. (*= identity; .= conservative substitution). D) Summary of syntenic relationships between human and mouse sequences. (* - proposed identification, ** - estimated size, ***- estimated size). **E)** Mitotic Localization of StARD9. A construct encoding A.A. 1-684 of human StARD9 (panels **A-D)** decorates the mitotic spindle (panel B) and overlaps with AurA labeling of the spindle poles (panel A). Fulllength StARD9 (panels E-L) incorporates into LAMP-1-positive lysosomes (panels E,H,I,L) during prometaphase (panels E-H) and anaphase (panels I-L). Mag. bar = 10 μm.

Journal of Cell Science • Supplementary information

Supplemental Figure 3.

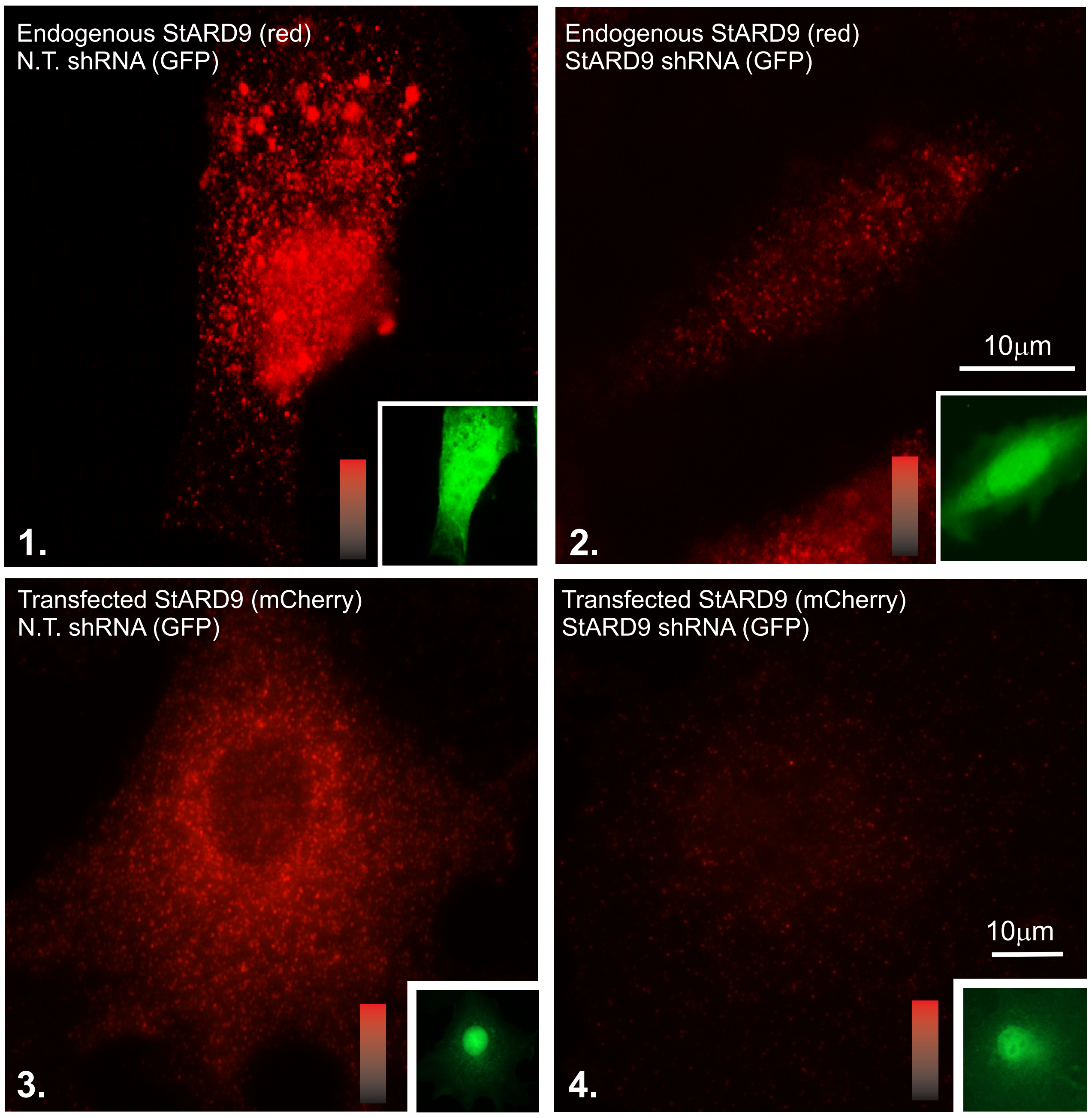

Fig. S3. ShRNA-based Depletion of StARD9. Immunofluorescence microscopy of endogenous StARD9 reveals the efficacy of the StARD9 shRNA (panel 2) compared to a non-targeting (N.T.) control (panel 1). ShRNA-expressing cells are identified by GFP-co-expression (see insets). Using a 4:1 ratio of shRNA:mCherry-StARD9, we also observed effective depletion of ectopically-expressed StARD9 in cells expressing the StARD9 shRNA (panel 4) compared to the non-targeting (N.T.) shRNA (panel 3). Intensity gradients reflect fluorescence intensities from 0-1200. Mag. bar = 10 μm.

Supplemental Figure 4.

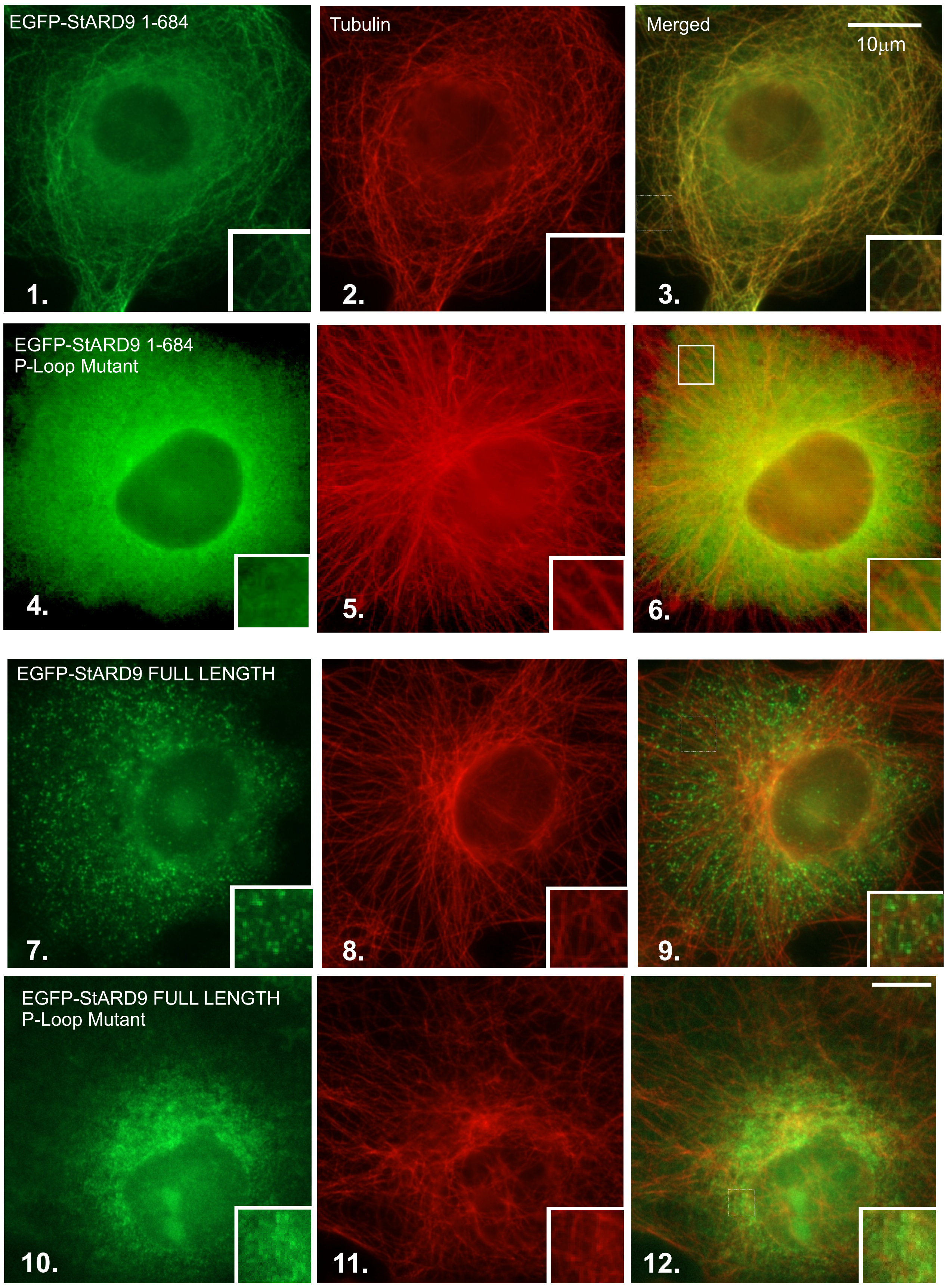

Fig. S4. Impact of StARD9 P-loop Mutations on Localization with Microtubules. Cells expressing a truncated StARD9 construct encoding A.A. 1-684 containing either wild-type (panels 1-3) or mutant P-loop sequence (panels 4-6) displayed differential colocalization with microtubules (panels 2&5). Cells expressing a full-length StARD9 construct containing either wild-type (panels 7-9) or mutant P-loop sequence (panels 10-12) displayed differential colocalization with microtubules (panels 8&11). Mag. bar = 10 μm.

Journal of Cell Science • Supplementary information

Supplemental Figure 5.

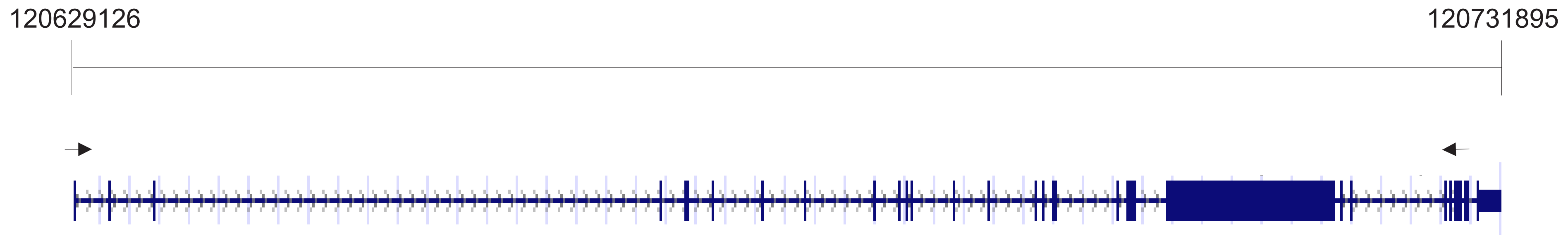

5′ END	GTGGCCGTGA**GGGTCCGGCCTCTCAGCAAG**	
		**GGTATAGCCAACTATACAGA**TGGCACCAGCCCAGGGTTGCCAGT 3′END
MOUSE		
F Het 2	GTGGCCGTGA**GGGTCCGG**	GCACCAGCCCAGGGTTGCCAGT
F Het 3	GTGGCCTTGAGGGTCCTG	GCACCAGTCCAGGGTTGCCAGT
F Het 6	GTGGCCGTGA**GGGTCCGG**	GCACCAGCCCAGGGTTGCCAGT
F Het 7	GTGGCCGTGA**GGGTCCGG**	GCACCAGCCCAGGGTTGCCAGT
M Het 13	GTGGCCGTGA**GGGTCCGG**	GCACCAGCCCAGGGTTGCCAGT
F Het 20	GTGGCCGTGA**GGGTCCGG**	GCACCAGCCCAGGGTTGCCAGT
M Het 28	GTGGCCGTGA**GGGTCCGG**	CACCAGCCCAGGGTTGCCAGT
F Het 32	GTGGCCGTGA**G**	**A**TGGCACCAGCCCAGGGTTGCCAGT
F Het 35	GTGGCCGTGA**GGGTCCGG**	CCCAGGGTTGCCAGT
M Het 47	GTGGCCGTGA**GGGTCCGGCCTCTCAG**	**A**TGGCACCAGCCCAGGGTTGCCAGT

Fig. S5. CRISPR/Cas9 Excision of StARD9 Gene. One-cell C57Bl/6 mouse embryos were injected with a cocktail of guide RNAs (arrows and bold sequences) that flank the coding region of the StARD9 gene (vertical lines identify exons in the StARD9 gene). 10/50 embryos displayed loss of at least one StARD9 allele. Sequencing of individual mouse lines revealed the newly-created junctional sequences of each line. Subsequent breeding and phenotypic analysis was focused on lines where genotyping was straightforward.

Journal of Cell Science • Supplementary information

Supplemental Figure 6.

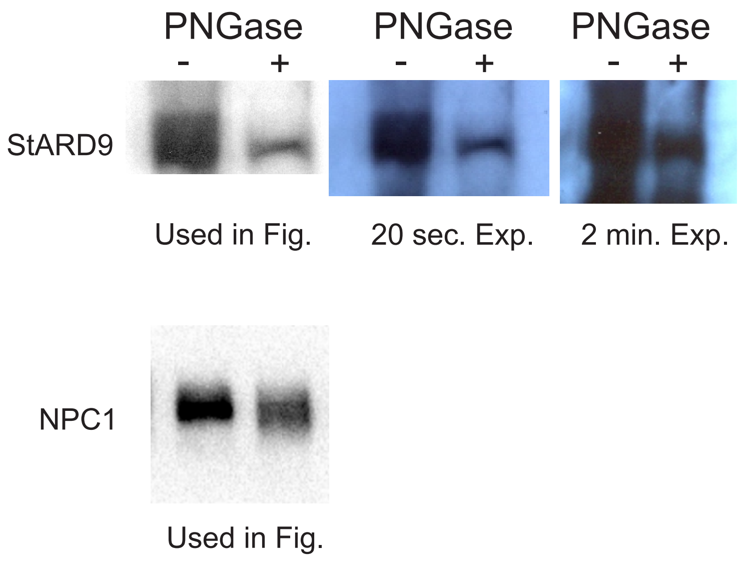

Fig. S6. Blot Transparency Figure. Multiple exposures of western blots for StARD9 (upper panels) and NPC1.

Journal of Cell Science • Supplementary information

Table S1. Proteomic analysis of phagosome membranes. (A) Purified phagosomes were subjected to extraction with PBS, 1 M NaCl or 200 mM NaCarbonate pH 11 followed by MRM analysis to detect tryptic peptides from StARD9, LAMP-1 and NPC1. The location (numbers) and A.A. sequence of peptides identified in this analysis are presented for each condition. No peptides were detected after detergent extraction (Fig. 2E). (B) Quantification of MRM analysis of indicated peptides under extraction conditions described above.

A.

PBS

StARD9

39-50	VDSRPESFGDTR
337-345	DSVLTWLLK
851-860	TLSTDCIPPK
1731-1740	IDSPQQTTTK
1892-1906	SLHCLSSPVIVAGGR
3885-3893	LDDSCVSEK

LAMP-1

315-325	ALQATVGNSYK
326-336	CNTEEHIFVSK

NPC1

43-52	YSGPPKPLPK
171-178	ALGLLCGR

NACL

StARD9

81-92	DLGTEVLSGAAK
337-345	DSVLTWLLK
851-860	TLSTDCIPPK
1081-1090	LLKPEDLGK
1731-1740	IDSPQQTTTK
1892-1906	SLHCLSSPVIVAGGR
2213-2224	TQGGVEEMTVDR
2299-2313	GVSSFEYILEPVMLK

LAMP-1

76-89	ENVSDPSLTITFGR
315-325	ALQATVGNSYK
326-336	CNTEEHIFVSK

NPC1

43-52	YSGPPKPLPK
171-178	ALGLLCGR

CARBONATE

StARD9

337-345	DSVLTWLLK
1081-1090	LLKPEDLQGK
1731-1740	IDSPQQTTTK
1892-1906	SLHCLSSPVIVAGGR
3885-3893	LDDSCVSEK

LAMP-1

90-99	GYLLTLNFTK
315-325	ALQATVGNSYK

NPC1

171-178	ALGLLCGR

B.

		PBS	1M NaCl	0.2 M NaCarbonate, pH 11	1% Tx-100, 1%SDS
**Protein**	**Peptide**	**Intensity (cps)**	**Intensity (cps)**	**Intensity (cps)**	**Intensity (cps)**
STARD9	R.DSVLTWLLK.E 2y5 337-345	70000.00	260000.00	50000.00	Undetectable
	R.SLH[C]LSSPVIVAGGR.S 2y11 1892-1906	27000.00	15000.00	15000.00	Undetectable
LAMP1	K.ALQATVGNSYK.[C] 2y8 315-325	7200.00	36000.00	35000.00	Undetectable
NPC1	K.ALGLLCGR.D 2y6 171-178	500.00	380.00	7000.00	Undetectable

Journal of Cell Science • Supplementary information

Table S2. Neuronal severity score of StARD9 (−/−) mice. (A) A composite score of tremors, ataxia, abnormal gait and hanging duration on the coat hanger test was used to compare StARD9 (+/+), (+/−) and (−/−) mice. StARD9 (-/-) mice progressed from healthy (***) to severely impaired (*) between P60 and P180. **(B)** Composite scores on the three genotypes (*n*=7 each) revealed the differences in neurodegeneration between wild-type StARD9 (+/+), StARD((+/−) and StARD9 mice at P120 and P180. Error bars = standard error and P-values were obtained from a Students t-test. *P*<0.005 (* and **; NS=not significant).

A.

Motor Coordination			
Genotype	P60	P120	P180
+/+	***	***	***
+/-	***	***	**
-/-	***	**	*

B.

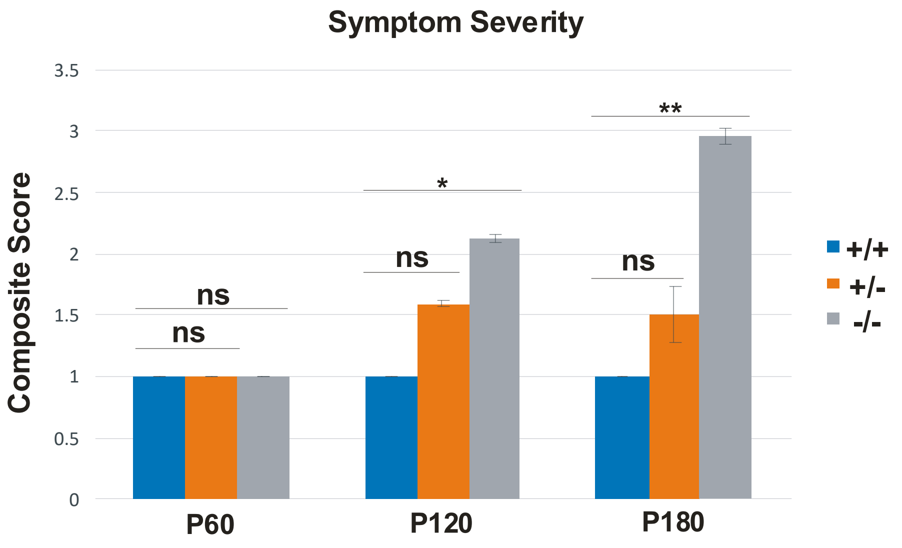

Table S3. Primers used for cDNA cloning and mutagenesis. The locations of primers used for RT-PCR cloning and PCR-based mutagenesis are indicated.

Primers Used for Cloning and Mutagenesis: Amino Acid Numbers	Forward primer	Reverse primer
STARD9: 1-240	ggttgtggcagatctatggcgaacgtgc	atcccattatctagaagaaaatacctaagg
STARD9: 240-520	ctgaaatggctagcaagatcaacc	ctgaccctgcaggacaatgtcctgttcctgg
STARD9: 520-680	ccaggaacaggacattgtcctgcaggg	ctggctaatccaagcttggtcaaattcc
STARD9: 680-1155	ggaatttgaccaagcttggattagc	ttggggctttggtacctcttctcagc
STARD9: 1155-1580	ctggctgagaagaggtaccaaagcccc	cataactcgcctctttctcactagtgc
STARD9: 1580-2135	gatttctttagcactagtgagaaagaggcg	cccaatgctgttaacctccatcgctcc
STARD9: 2135-2919	ggagcgatggaggttaacagcattggg	ttcccttgggtgtctacatggggcttcc
P-loop Mutation (STARD9)	gacaggctctgggatcgcatataccatgctgg	tgtccataagcaaaaaggcatatgttatagc c
shRNA Silent (STARD9)	aaagaaagatgcaaccagaacaccttcctcagct gatcc	ttctctatgatctccgaagagctcgagtggca tataaatgg

Journal of Cell Science • Supplementary information

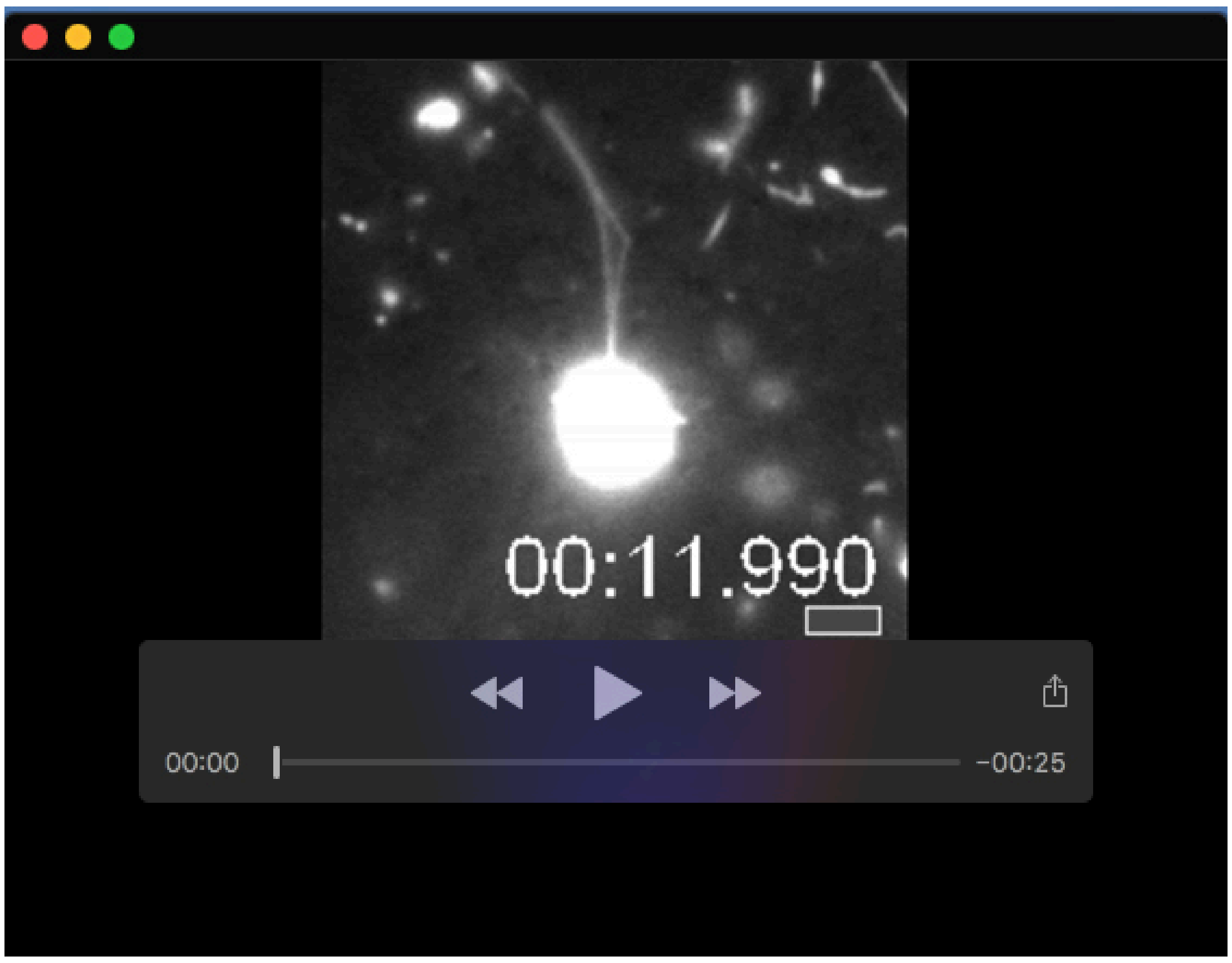

Movie 1. Live-cell imaging of a COS-7 cell expressing wild-type NPC1-EGFP (white), highlighting a single lysosome and the projection of a membrane tubule from the lysosome surface. Mag. bar = 2 µm and time stamp is mins:secs:millisecs.

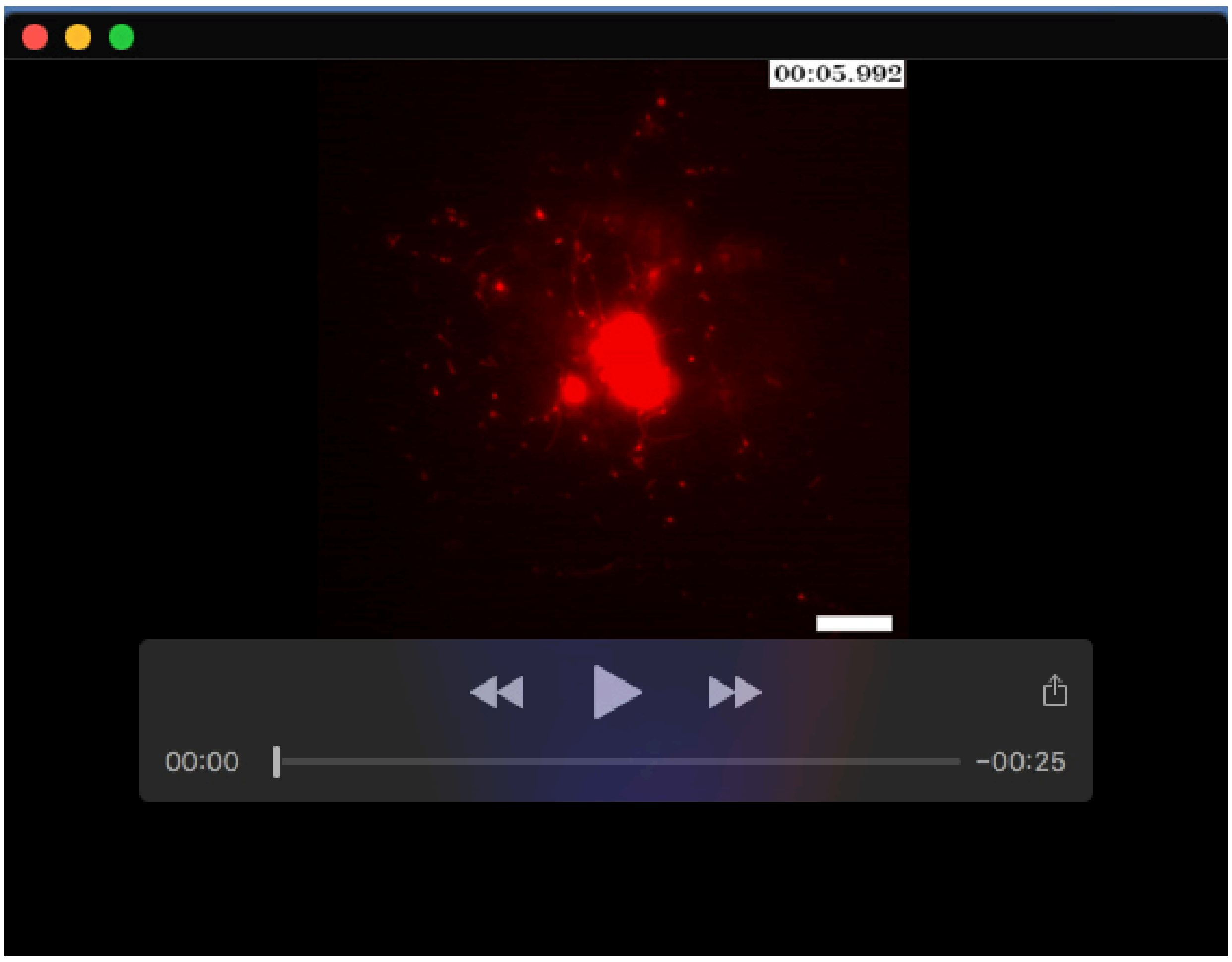

Movie 2. Live-cell imaging of a COS-7 cell expressing CathepsinB-mCherry (red), highlighting the projection of membrane tubules from LE/Ls. Mag. bar = 10 µm and time stamp is mins:secs:millisecs.

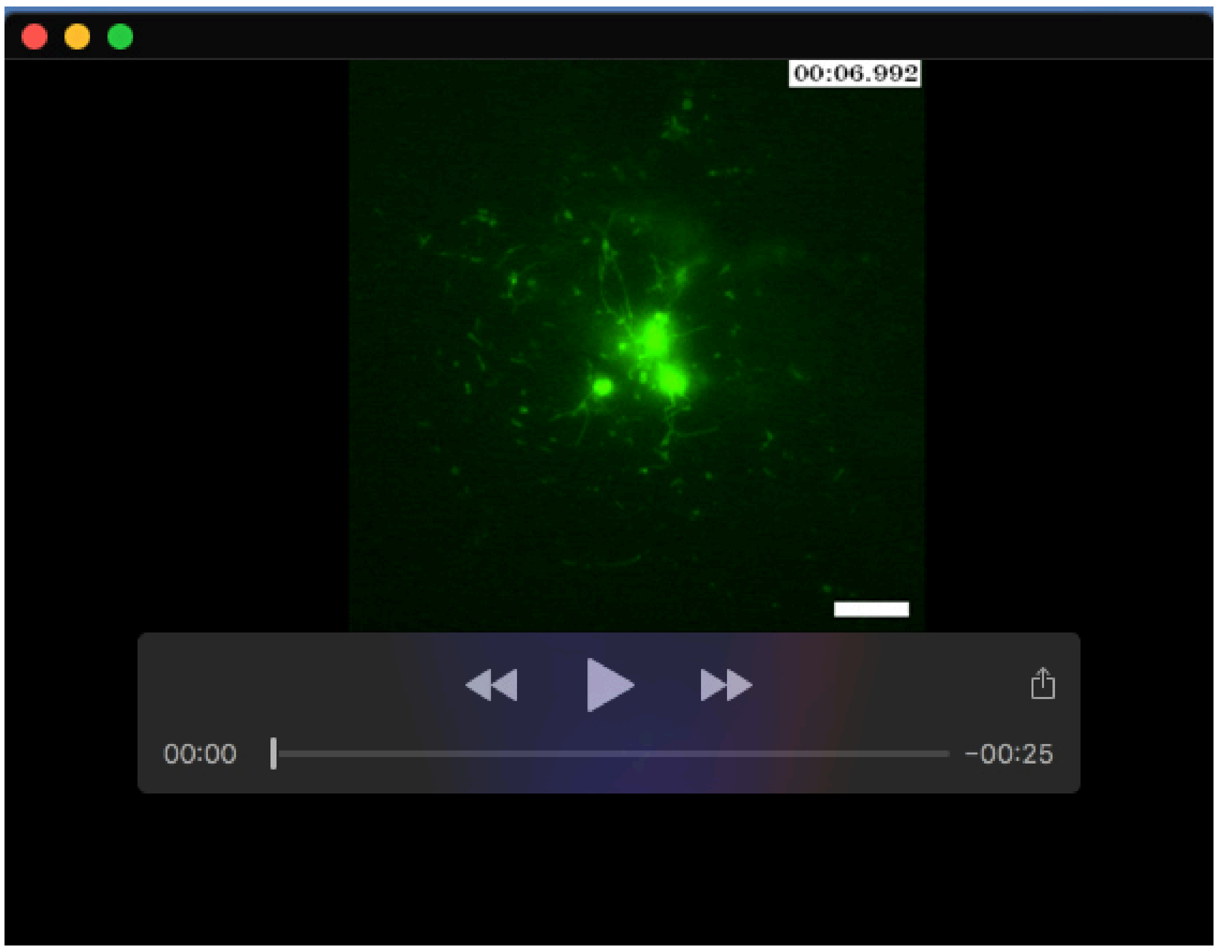

Movie 3. Live-cell imaging of a COS-7 cell expressing NPC1-EGFP (green), highlighting the projection of membrane tubules from LE/Ls. Mag. bar = 10 m and time stamp is mins:secs:millisecs.

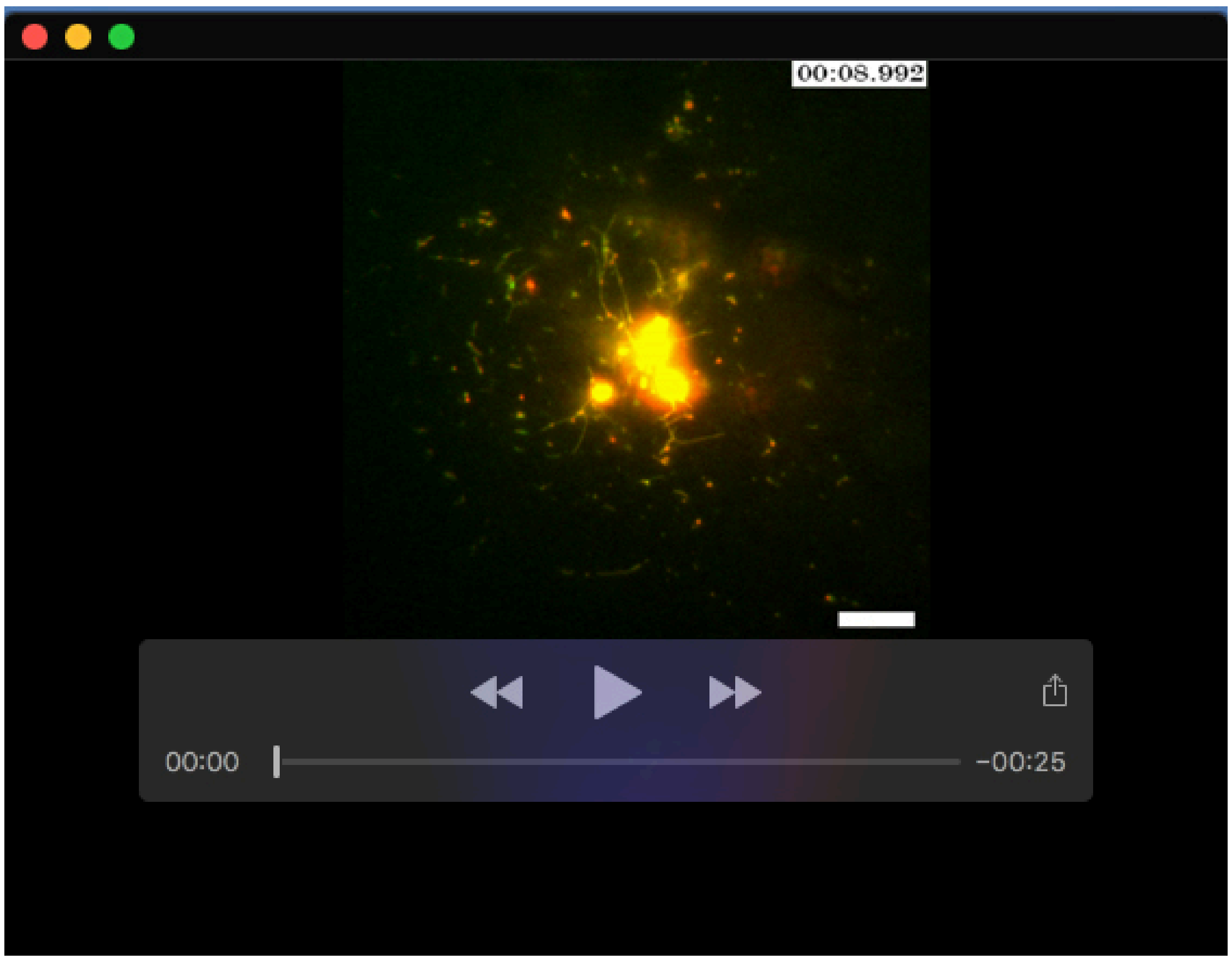

Movie 4. Merged image of live-cell imaging of a COS-7 cell co-expressing CathepsinB-mCherry (red) and NPC1-EGFP (green), highlighting the colocalization of both markers in the same membrane tubules. Mag. bar = 10 μm and time stamp is mins:secs:millisecs.

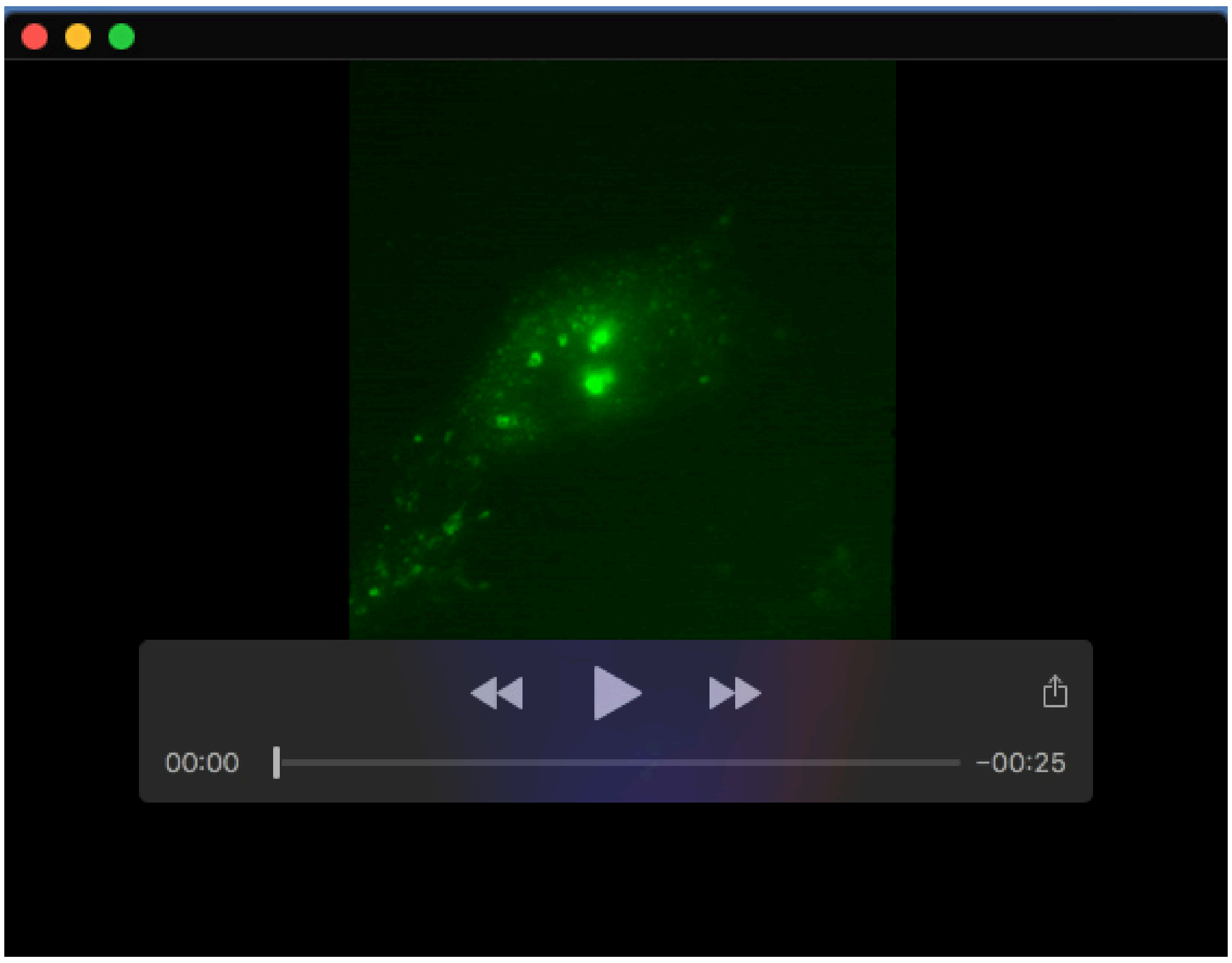

Movie 5. Live-cell imaging of a COS-7 cell expressing I1061T mutant NPC1-EGFP (white), demonstrating a loss of LE/L motility and tubulation. Mag. bar = 5 µm and time stamp is mins:secs:millisecs.

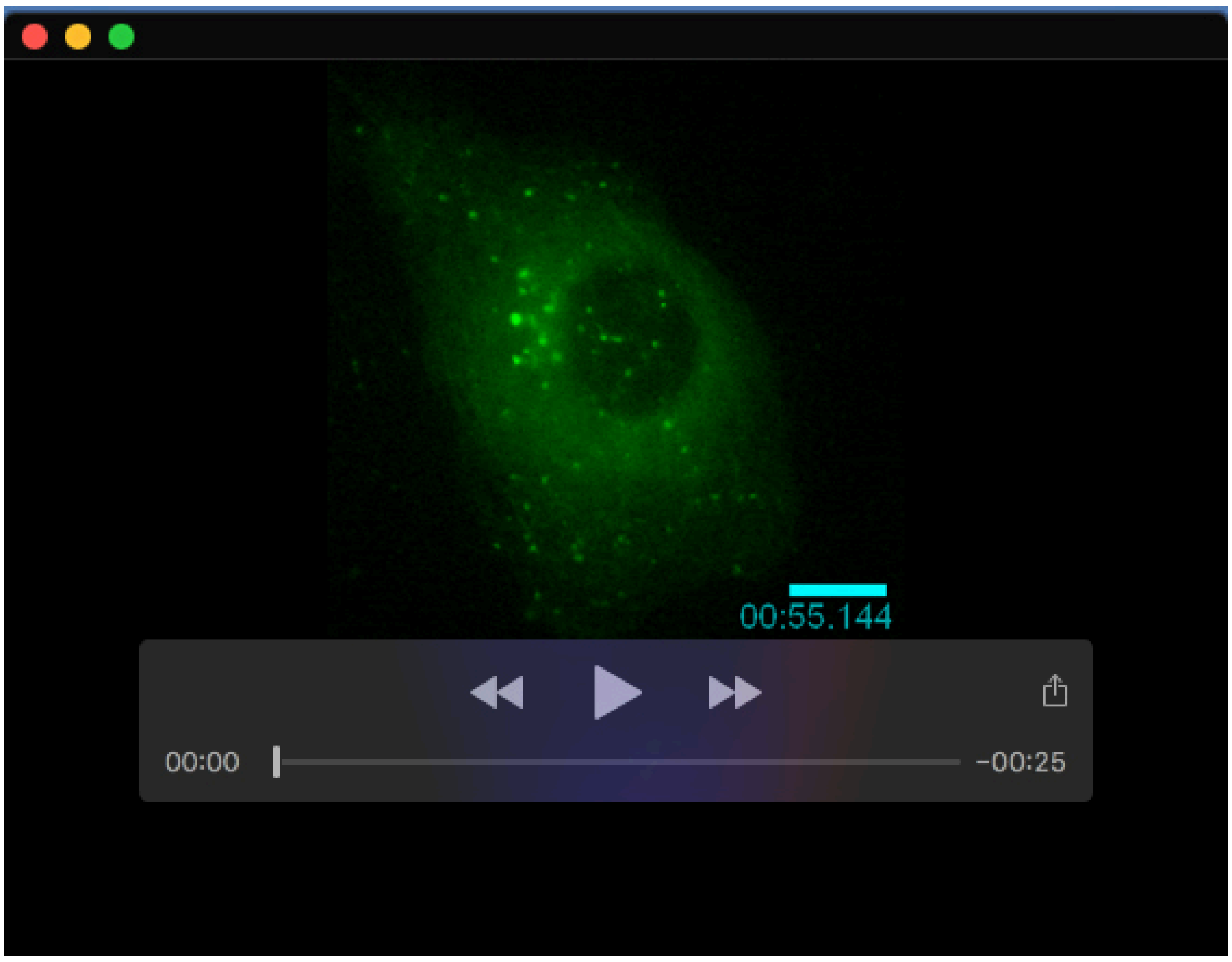

Movie 6. Live-cell imaging of a COS-7 cell expressing EGFP-StARD9 (green). Mag. bar = 5 µm and time stamp is mins:secs.millisecs.

Journal of Cell Science • Supplementary information

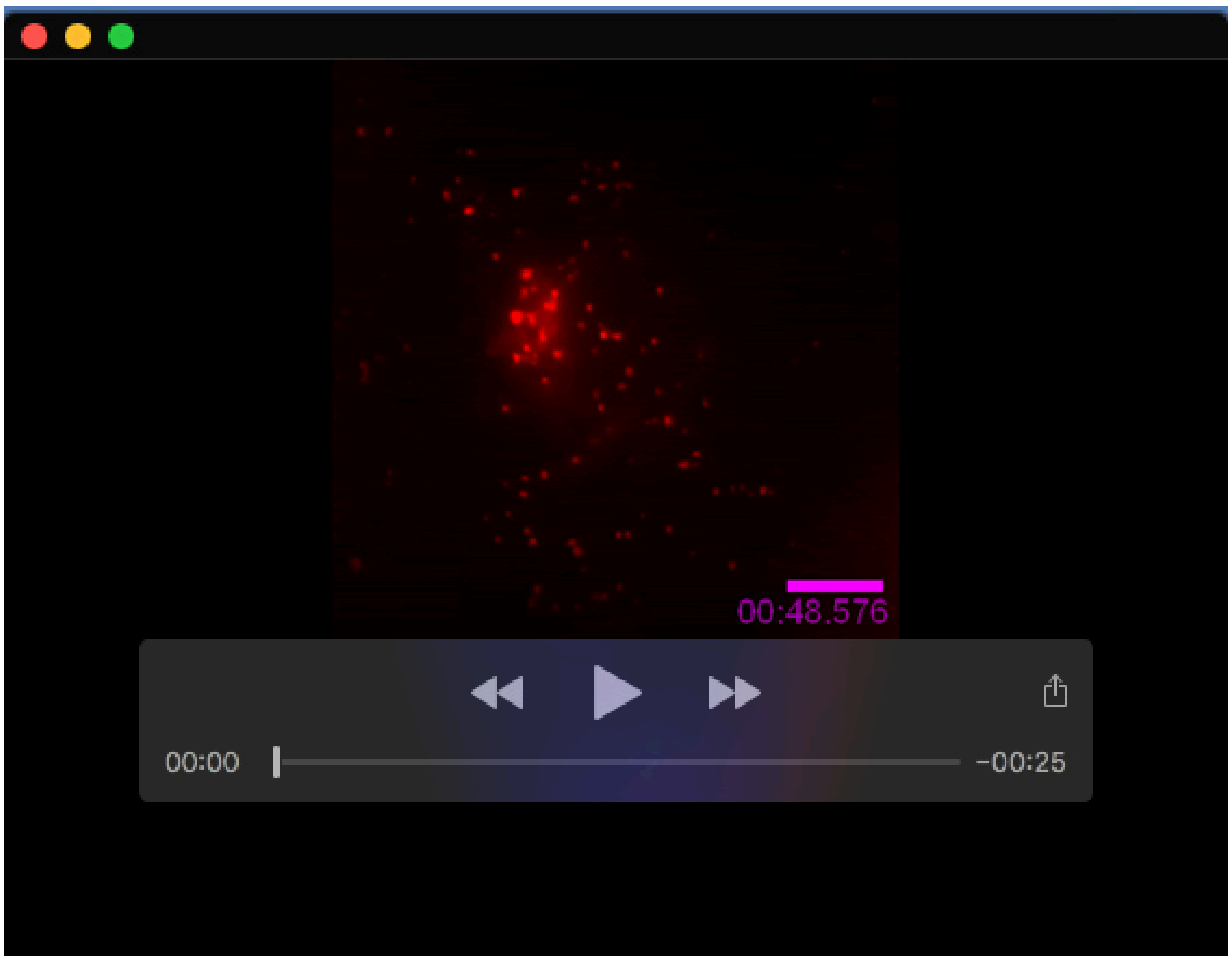

Movie 7. Live-cell imaging of a COS-7 cell labeled with Lysotracker. Mag. bar = 5 µm and time stamp is mins:secs.millisecs.

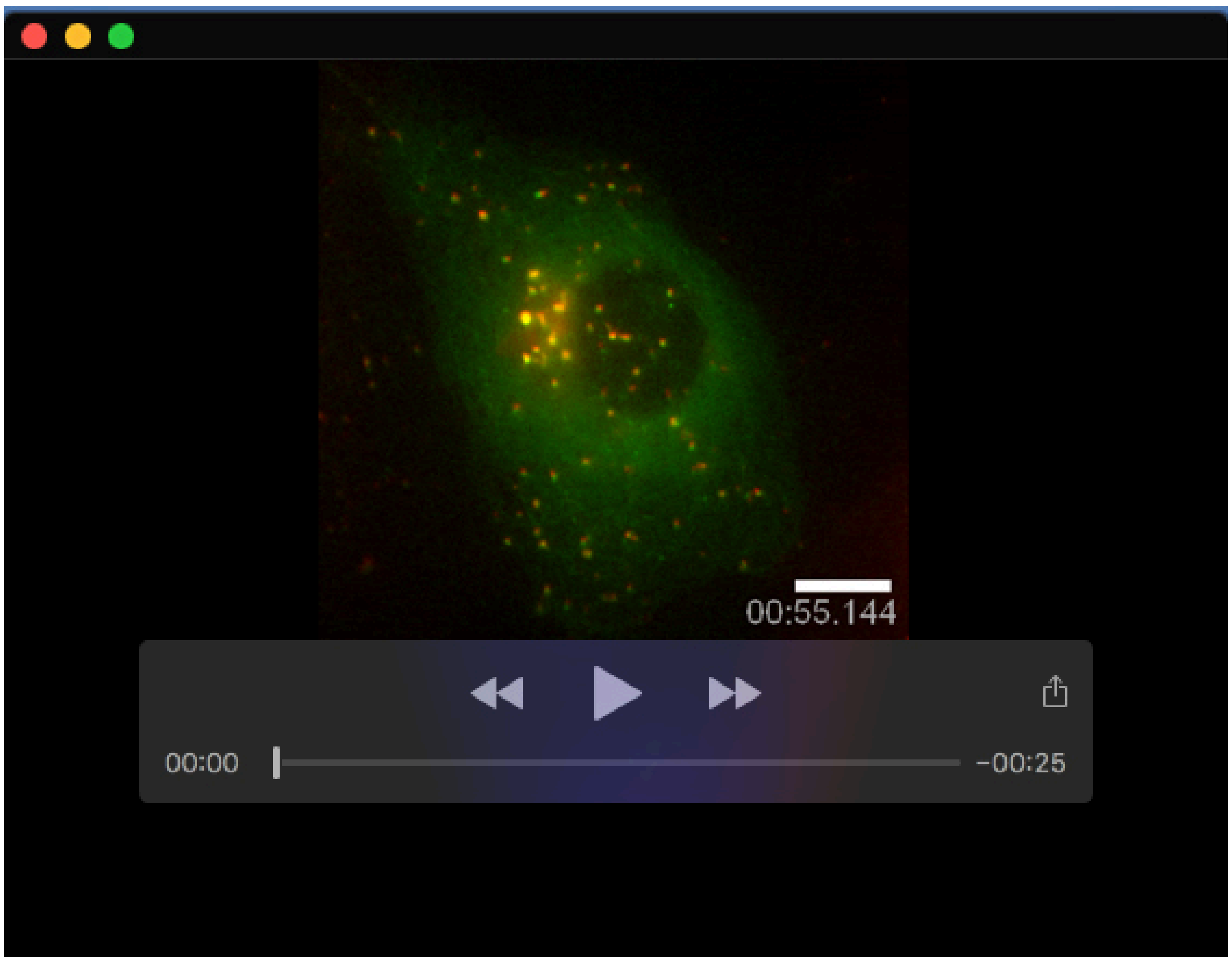

Movie 8. Merged images of a COS-7 cell expressing full-length EGFP-StARD9 (green) and labeled with Lysotracker (red). Mag. bar = 5 µm and time stamp is mins:secs.millisecs.

Journal of Cell Science • Supplementary information

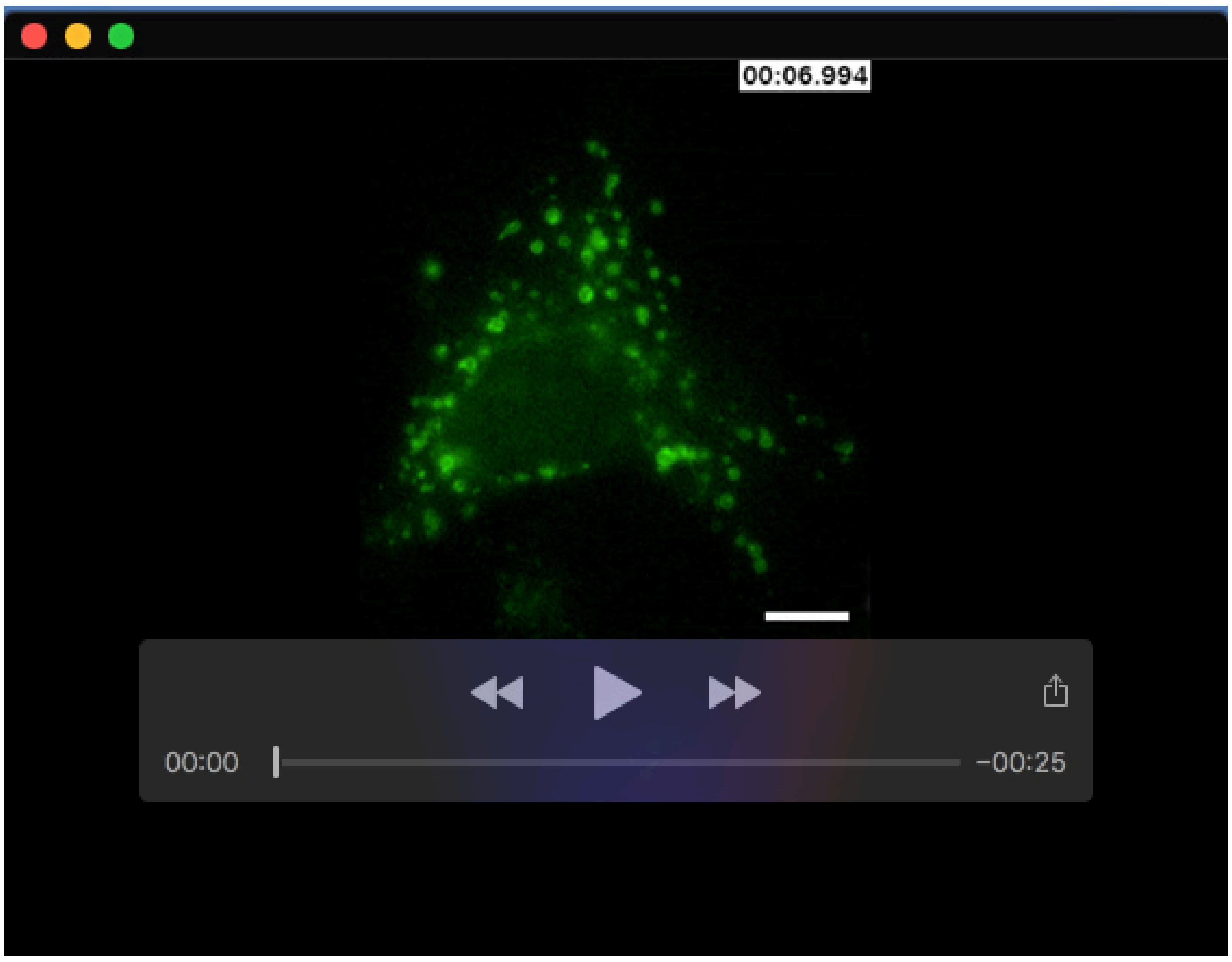

Movie 9. Live-cell imaging of a COS-7 cells expressing NPC1-EGFP (green). Mag. bar = 5 μm and time stamp is mins:secs.millisecs.

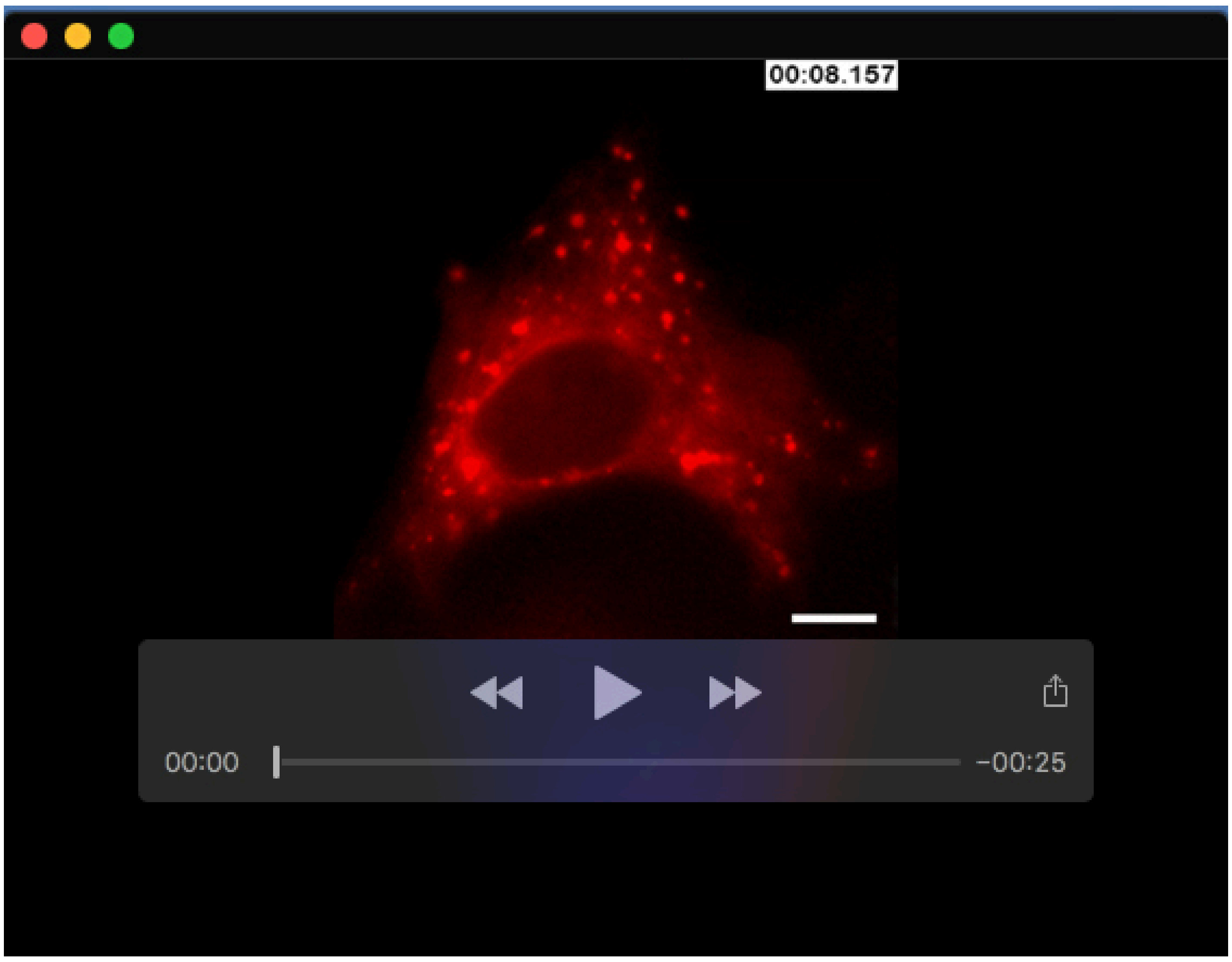

Movie 10. Live-cell imaging of a COS-7 cells expressing mCherry-StARD9 (red). Mag. bar = 5 μm and time stamp is mins:secs.millisecs.

Journal of Cell Science • Supplementary information

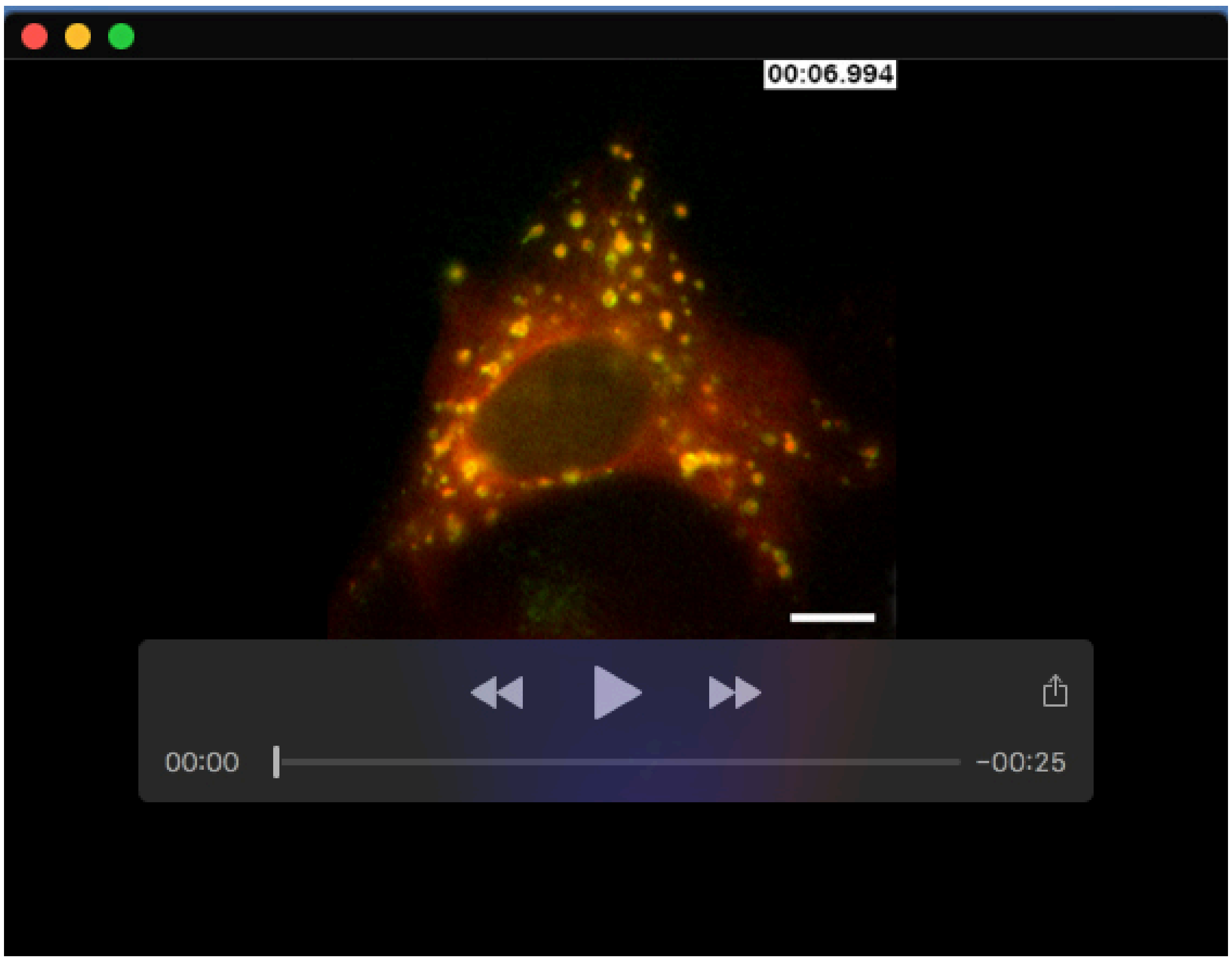

Movie 11. Merged images of a COS-7 cells co-expressing NPC1-EGFP (green) and mCherry-StARD9 (red). Colocalization is highlighted in yellow. Mag. bar = 5 μm and time stamp is mins:secs.millisecs.

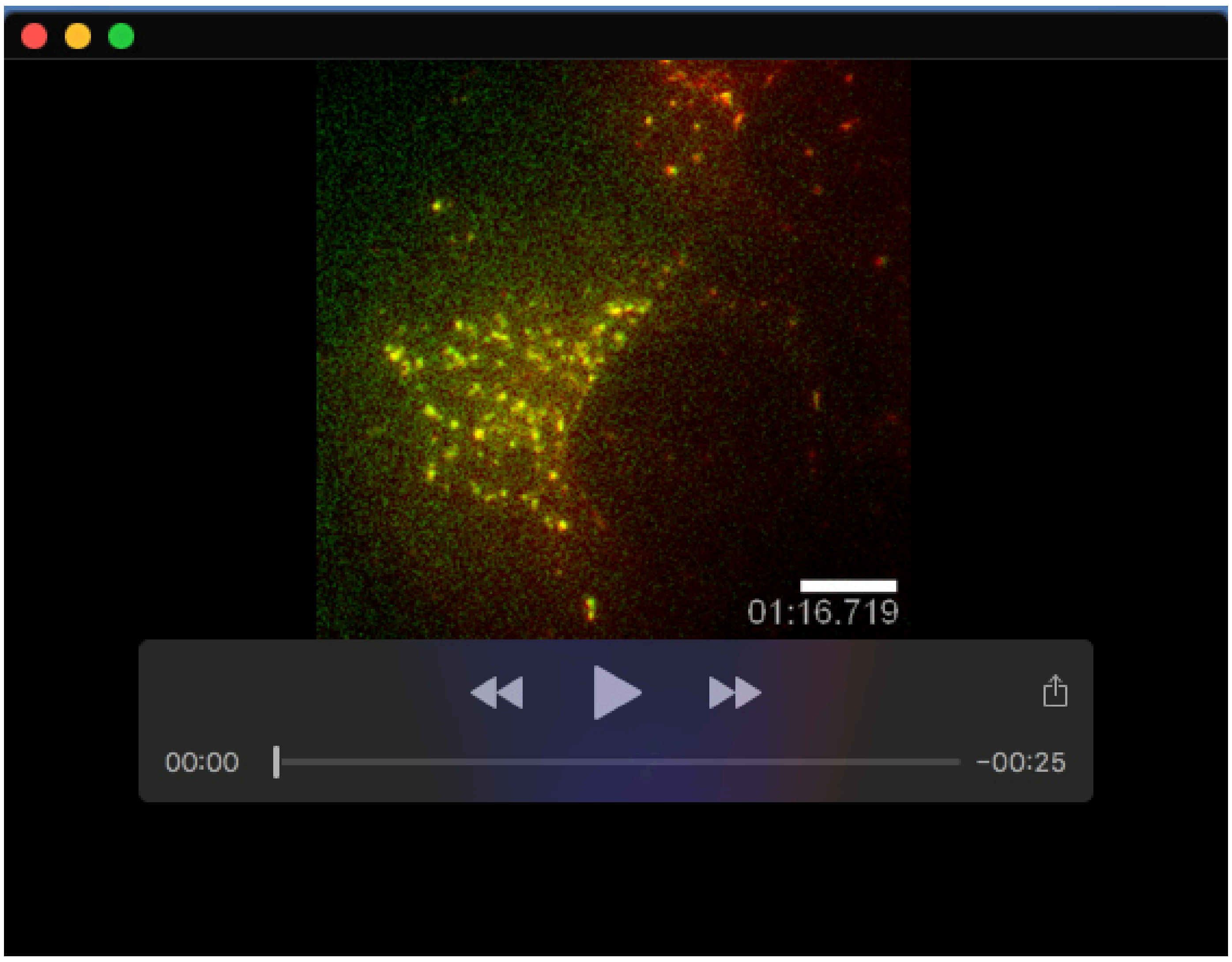

Movie 12. Merged images of a COS-7 cells co-expressing EGFP-StARD9 P-loop Mutant (green) and stained for Lysotracker (red). Colocalization is highlighted in yellow. Mag. bar = 5 μm and time stamp is mins:secs.millisecs.

Journal of Cell Science • Supplementary information
